# The Burden of Nonalcoholic Steatohepatitis: A Systematic Review of Epidemiology Studies

**DOI:** 10.1016/j.gastha.2022.06.016

**Published:** 2022-07-19

**Authors:** Elliot B. Tapper, Charlotte Fleming, Adriana Rendon, João Fernandes, Pierre Johansen, Margarida Augusto, Sunita Nair

**Affiliations:** 1 Division of Gastroenterology and Hepatology, University of Michigan, Ann Arbor, Michigan, USA; 2 Clarivate, Bicester, UK; 3 Global Medical Affairs, Novo Nordisk A/S, Søborg, Denmark; 4 Payer Evidence Generation, Novo Nordisk A/S, Søborg, Denmark; 5 Novo Nordisk Denmark A/S, Region North & West Europe, Ørestad, Denmark; 6 Novo Nordisk Ltd, Gatwick, UK; 7 Clarivate, Mumbai, India

**Keywords:** Liver Transplantation, Mortality, Progression, Risk Factors

## Abstract

**Background and Aims:**

Nonalcoholic steatohepatitis (NASH) is associated with increased mortality and risk of complications but is often asymptomatic and under-recognized. A systematic review of NASH epidemiology was conducted to provide information on the burden of NASH and highlight important evidence gaps for future research.

**Methods:**

Medline, EMBASE, and Cochrane Library databases were searched for English-language publications published from 2010 to January 2022 that reported on natural history, risk factors, comorbidities, and complications of a NASH population or subpopulation.

**Results:**

Overall, 173 publications were included. NASH was shown to have a variable disease course and high prevalence of comorbid disease. Although many patients progressed to cirrhosis and end-stage liver disease, disease regression or resolution was reported in up to half of patients in some studies. Reported risk factors for disease progression or resolution included levels of (or changes in) serum fibrosis markers, liver enzymes, and platelets. The presence of NASH increased the risk of liver cirrhosis and other serious diseases such as hepatocellular carcinoma, colorectal cancer, chronic kidney disease, and cardiovascular disease. In 2017, NASH was responsible for ∼118,000 cirrhosis deaths globally, and an increasing proportion of patients are receiving liver transplantation for NASH in Europe and the United States. Consolidation of data was hampered by heterogeneity across the studies in terms of patient populations, follow-up time, and outcomes measured.

**Conclusion:**

NASH is associated with significant morbidity and mortality, an increased risk of comorbidities, and imposes an increasing burden among liver transplantation recipients. Longer studies with harmonized study criteria are required to better understand the impact of NASH on patient outcomes.

## Introduction

Nonalcoholic fatty liver disease (NAFLD) is one of the most common causes of chronic liver disease worldwide, with an estimated global prevalence of 25%.^1^ NAFLD represents a spectrum of liver disease ranging from simple steatosis to nonalcoholic steatohepatitis (NASH), which can then progress to cirrhosis.^2^ NASH, the most severe form of NAFLD, occurs when excess fat has accumulated in the liver, and lobular inflammation and hepatocellular ballooning are present.^3^ Despite this, NASH is often perceived as asymptomatic or unrecognized and may not be diagnosed until late stages.^4^^,^^5^

NASH occurs in almost one-quarter of patients with NAFLD.^6^ It has an estimated prevalence of 1.50%–6.45% in the general population,^1^ although disease prevalence and incidence estimates vary considerably due to differing patient populations and disease criteria.^2^ NASH is associated with obesity and type 2 diabetes (T2D), hepatic complications including cirrhosis, hepatocellular carcinoma (HCC), and extrahepatic conditions including cardiovascular (CV) disease (CVD) and cancer.^3^^,^^7^ NASH cirrhosis is predicted to become the leading cause of liver transplantation (LT) in the United States between 2020 and 2025.^8^

This study aimed to summarize and critically appraise studies on the natural history of NASH, risk factors for NASH, hepatic and extrahepatic complications associated with NASH, related comorbidities, and LT outcomes, and to highlight important evidence gaps for future research.

## Methods

A systematic literature review was conducted using Medline, EMBASE, and Cochrane Library databases via the Ovid platform using predefined search strategies (Table A1). The review was conducted in line with Cochrane guidelines^9^ and is reported in line with Preferred Reporting Items for Systematic Reviews and Meta-Analyses guidelines.^10^

The primary systematic literature review was conducted in February 2020, with updates in January 2021 and January 2022 (last searches ran on January 17, 2022). Relevant conference proceedings from 2017 to February 10, 2020, were handsearched for unpublished studies (repeated on January 6, 2021, and January 17–18, 2022), and submission documents from Health Technology Assessment agencies were reviewed for relevant data (Table A2). Conference abstracts were identified and screened, but data were not extracted as it was anticipated that the large number of full-text publications would provide an adequate evidence base of relevant information. Eligible publications were full-text English-language epidemiology studies that included outcomes in NASH populations (with or without comorbidities), or a NASH subgroup within a NAFLD study, published from 2010 to January 2022. Data from NAFLD or heterogeneous NAFLD/NASH populations were excluded. Full eligibility criteria are described in Table A3.

First-round screening of titles and abstracts was followed by second-round full-text screening of short-listed articles and data extraction from articles meeting the eligibility criteria. Both first- and second-round screenings were performed by 2 independent researchers, and final inclusion was verified by the project lead. Data extraction was performed using predesigned data extraction tables in Microsoft® Excel by an analyst with a 100% quality check by an independent senior analyst. Data extracted from each study included, but were not limited to, reference, country, study design, publication and study characteristics, baseline characteristics, intervention(s), and outcomes reported. Disputes regarding eligibility or data extraction were referred to a third party.

The quality of included observational studies was assessed using the Quality Assessment Tool for Quantitative Studies produced as part of the Effective Public Health Practice Project.^11^ This assesses the quality of 6 components to assign a global rating for each study of “strong” (no weak ratings for any of the listed criteria), “moderate” (one weak rating), or “weak” (2 or more weak ratings). The components are selection bias (likelihood of representativeness of target population and percentage participation), design (study type), confounders (percentage of relevant confounders controlled for), blinding (of outcome assessors and study participants), data collection methods (validity and reliability), and withdrawals and dropouts. Any component that did not apply to the study was marked “not applicable.” Quality assessment (risk of bias) of randomized controlled trials was conducted using the 7-criteria checklist provided in Section 4.6 of the UK National Institute for Health and Care Excellence single technology appraisal user guide to assess the likelihood of selection, performance, attrition, and detection bias.^12^ Quality assessments were not used to exclude studies. Two reviewers independently assessed the likelihood of bias, and any disagreements were resolved by discussion and/or additional referees.

## Results

The electronic database searches identified 12,105 citations, of which 2022 were identified as duplicates and excluded. A further 9926 were excluded after screening by title and abstract or full-text screening. Sixteen studies that met the eligibility criteria were identified from handsearching, resulting in inclusion of 173 publications covering 167 unique studies (Figure A1 and Table A4). An overview of the study and patient characteristics across the identified studies is included in Table 1, Table 2, Table 3, Table 4, Table 5, Table 6, Table 7 and Tables A5–A7.

### Disease Trajectory

Only 6 studies reported on aspects of disease progression or regression that occurred naturally over time without pharmacological intervention (Table 1). Study populations and outcomes measured were heterogeneous, and it was therefore not possible to make direct comparisons across studies. Generally, further disease progression was common among patients with advanced disease at baseline.^13^, ^14^, ^15^, ^16^ Rates of progression to cirrhosis were shown to increase with increasing fibrosis severity in a Spanish database analysis,^16^ and a 22% rate of progression from F3 fibrosis to cirrhosis over a median 29 months was reported in a pooled analysis of the phase 2b simtuzumab trials (in which simtuzumab is assumed to have no biological activity).^15^ Interestingly, patient age was linked to disease progression, but not regression, in a study of first-time adult LT registrants; in this study, removal from the LT wait list because of death or being too sick increased with increasing age, whereas removal from the LT wait list due to condition improving was similar regardless of patient age.^14^

**Table 1:** Progression or Regression of NASH and Fibrosis Over Time

Reference	Country/region	Data source/population	Study type	N	Disease progression
O'Leary et al^13^	USA	NASH/CC or HCV patients listed for an orthotopic LT (single center, 2002–2008)	Observational cohort study	NASH/CC: 217 HCV: 645	• Median baseline MELD score of 14 • 5-y follow-up • In patients with MELD score ≤15, median rate of progression of 1.3 MELD points per year • Rate of progression could not be calculated in patients with MELD score >15 as generally received LT within 1 y
Ajmera et al^17^	USA	NASH CRN participants ≥21 y with biopsy-confirmed NAFLD and alcohol use history within 2 y of initial biopsy^a^	Observational cohort study	NASH: 182 Borderline NASH: 49 NAFL: 54	• Mean 47 (± 26) mo between paired biopsies for mixed population • Among patients with definite NASH, consistent nondrinkers^b^ were most likely to have NASH resolution: ○ Consistent nondrinkers: 22% ○ Modest drinker to nondrinker: 17% ○ Nondrinker to modest drinker: 13% ○ Consistent modest drinker: 11% • In logistic regression models adjusted for sex, age, race, and smoking history, consistent modest drinkers were significantly less likely to have NASH resolution than consistent nondrinkers (adjusted OR 0.32, 95% CI 0.11–0.92, *P* = .04)
Reddy et al^18^	USA	Electronic medical records of patients with biopsy-proven NAFLD with ≥2 liver biopsies ≥1 y apart (2006–2016)	Observational chart review study	NASH: 10 NAFL: 26	• Median 3.8 y between paired biopsies for mixed population • At follow-up, 7 patients with NAFL progressed to NASH and 5 patients with NASH no longer met NASH criteria (NAS ≥5) • There was a 50% fibrosis progression in patients with NASH: ○ At baseline, 80% of patients had a fibrosis score of ≤2, and 20% had a fibrosis score of 3/4 ○ On follow-up, 58% of patients had a fibrosis score of ≤2, and 42% had a fibrosis score of 3/4
Henson et al^14^	USA	First-time adult LT registrants (UNOS database, 2004–2017)	Retrospective database analysis	NASH: 14,197 ALD: 27,053 HCV: 24,827	• In NASH patients, removal from the LT wait list because of death or being too sick increased with increasing age: ○ 18.8%, 22.5%, 26.3%, 27.3%, 31.4%, and 36.1% in 18–49, 50–54, 55–59, 60–64, 65–69, and ≥70 y, respectively • Removal from the LT wait list due to condition improving was similar regardless of patient age: ○ 5.0%, 3.1%, 2.6%, 2.8%, 3.6%, and 4.1% in 18–49, 50–54, 55–59, 60–64, 65–69, and ≥70 y, respectively
Sanyal et al^15^	Global	Patients with biopsy-confirmed NASH and bridging fibrosis (F3) or compensated cirrhosis (F4)^c^	Interventional study (Phase 2b RCTs^d^)	NASH with F3 fibrosis: 217 NASH with compensated cirrhosis: 258	• After a median follow-up of 29.0 mo (range 0.3–46.3), progression to cirrhosis occurred in 22% of patients with F3 fibrosis
Ampuero et al^16^	Spain	Patients with biopsy-proven NAFLD (HepaMet registry)	Retrospective database study	Survival analysis: NAFL: 281 Indeterminate NASH: 355 NASH: 598	• Mean 4.7 ± 3.8 y follow-up for full NAFLD cohort^e^ • Despite having more patients with advanced liver disease, survival and rate of first cirrhosis decompensation were similar for patients with NASH vs indeterminate NASH or NAFL, respectively: ○ Survival: 2.7% vs 1.4% vs 4.6% ○ Rate of first cirrhosis decompensation: 2% vs 1.7% vs 2.1% • Progression to cirrhosis was significantly higher for NASH and indeterminate NASH vs NAFL (9.5% vs 7.9% vs 5%, respectively); this translated to a higher annual incidence of progression to cirrhosis with NASH and indeterminate NASH vs NAFL • Annual incidence of progression to cirrhosis increased with increasing fibrosis severity

ALD, alcoholic liver disease; CC, cryptogenic cirrhosis; CI, confidence interval; CRN, Clinical Research Network; HCV, hepatitis C virus; LT, liver transplantation; MELD, Model for End-Stage Liver Disease; NAFL, nonalcoholic fatty liver; NAFLD, nonalcoholic fatty liver disease; NAS, NAFLD Activity Score; NASH, nonalcoholic steatohepatitis; OR, odds ratio; RCT, randomized controlled trial; UNOS, United Network for Organ Sharing.

a Participants were drawn from 3 groups: (1) the adult NAFLD Database study, (2) adults on placebo in the Pioglitazone vs Vitamin E vs Placebo for the Treatment of Non-Diabetic Patients with Nonalcoholic Steatohepatitis (PIVENS) trial, and (3) adults on placebo in the Farnesoid × Receptor Ligand Obeticholic Acid in NASH Treatment (FLINT) trial.

b Nondrinkers were defined as current “never” drinkers who have not, over the course of their lifetime, had ≥1 alcoholic drink/month during a 12-month period or had ≥3 drinks per day for ≥3 consecutive days, whereas modest drinkers were defined as those drinking ≤2 drinks on a typical day, in the absence of monthly (or more frequent) heavy drinking.

c Simtuzumab- and placebo-treated patients were pooled based on the assumption that simtuzumab has no biological activity, supported by its failure to improve liver histology, portal pressure, liver biochemistry, and serum fibrosis markers in RCTs.

d NCT01672866 and NCT01672879.

e Excluding patients who underwent liver biopsy during bariatric surgery (n = 539) or were enrolled in clinical trials (n = 120).

Nineteen interventional studies (over 21 publications) reported on the reversibility of histologic changes associated with NASH and fibrosis stage in response to therapy, lifestyle intervention, or placebo (summarized in Table 2). Histologic improvement in NASH (NAFLD Activity Score [NAS] improvement of ≥1 or ≥2 stages or improvement in Steatosis, Activity, and Fibrosis score) were reported in up to 92% of patients receiving pharmacologic therapies, with the highest rates of improvement seen with efruxifermin and semaglutide.^33^^,^^37^ Histologic improvement was also seen in 47%–61% of patients receiving lifestyle intervention,^20^^,^^25^ whereas improvement in placebo or control arms generally occurred in one-third or fewer participants.^20^^,^^21^^,^^26^^,^^28^^,^^30^^,^^32^^,^^33^^,^^36^, ^37^, ^38^, ^39^ Significantly greater proportions of patients achieving resolution of NASH (or resolution of NASH without worsening of fibrosis) compared with placebo were reported for few pharmacologic interventions. These included vitamin E (36% vs 21%, *P* = .05), pioglitazone (47% vs 21%, *P* < .001 and 51% vs 19%, *P* < .001), elafibranor (19% vs 12%, *P* = .045), and semaglutide 0.4 mg (59% vs 17%, *P* < .001).^21^^,^^26^^,^^27^^,^^37^ NASH resolution was seen in up to 22% of patients receiving placebo or control.^21^^,^^26^, ^27^, ^28^^,^^30^^,^^32^^,^^34^^,^^36^^,^^37^^,^^39^ Bariatric surgery was the most effective intervention in terms of NASH improvement, with NASH disappearing in 85% of recipients.^23^ In some studies, improvements in NASH were accompanied by improvements in fibrosis,^23^^,^^24^^,^^27^^,^^30^^,^^33^ but improvements in NASH without substantial improvement in fibrosis were also reported in several studies,^19^, ^20^, ^21^^,^^34^^,^^37^ and one study reported improvements in fibrosis without improvements in NAS.^28^

**Table 2:** Changes in NASH and Fibrosis in Response to Interventions

Reference	Country	Data source/population	Study type	N	Intervention	Duration	Effects on NASH	Effects on fibrosis
Kimura et al^19^	Japan	Patients with biopsy-proven NASH and dyslipidemia	Single-arm open-label cohort study	43	Atorvastatin	1 y	NAS improved: 68% No change: 27% Exacerbated: 5%	Improved: 9% No change: 59% Exacerbated: 32%
Promrat et al^20^	USA	Physician-recruited patients with biopsy-confirmed NASH from Rhode Island area	Interventional study (RCT)	31	Lifestyle vs control (basic education)	48 wk	**Mean change in NAS score:** Lifestyle vs control: −2.4 vs −1.4, *P* = .05 **NAS improvement ≥3 points:** Lifestyle vs control: 61% vs 20%, *P* = .04	**Mean change in fibrosis score:** Lifestyle vs control: 0.0 vs −0.3, *P* = .62
Sanyal et al^21^	USA	Nondiabetic patients with biopsy-confirmed NASH (PIVENS; NCT00063622)	Interventional study (RCT)	247	Vitamin E 800 IU vs pioglitazone 30 mg vs PBO	96 wk	**Histologic improvement in NASH:**^a^ Vitamin E vs PBO: 43% vs 19%, *P* = .001 Pioglitazone vs PBO: 34% vs 19%, *P* = .04 **Mean change in NAS:** Vitamin E vs PBO: −1.9 vs −0.5, *P* < .001 Pioglitazone vs PBO: −1.9 vs −0.5, *P* < .001 **Resolution of definite NASH:** Vitamin E vs PBO: 36% vs 21%, *P* = .05 Pioglitazone vs PBO: 47% vs 21%, *P* < .001	**Fibrosis improvement:** Vitamin E vs PBO: 41% vs 31%, *P* = .24 Pioglitazone vs PBO: 44% vs 31%, *P* = .12 **Mean change in fibrosis score:** Vitamin E vs PBO: −0.3 vs −0.1, *P* = .19 Pioglitazone vs PBO: −0.4 vs −0.1, *P* = .10
Hoofnagle et al^22^	USA	Nondiabetic patients with biopsy-confirmed NASH (PIVENS; NCT00063622)	Interventional study (RCT)	139	Vitamin E 800 IU vs PBO	96 wk	**Mean change in NAS:** Vitamin E – ALT responders vs nonresponders:^b^ −3.0 vs −0.8, *P* < .001 PBO – ALT responders vs nonresponders: −1.6 vs −0.4, *P* = .03 **≥2-point reduction in NAS:** Vitamin E – ALT responders vs nonresponders: 82% vs 32%, *P* < .001 PBO – ALT responders vs nonresponders: 55% vs 25%, *P* = .07	**Mean change in fibrosis score:** Vitamin E – ALT responders vs nonresponders: −0.5 vs −0.2, *P* = .34 PBO – ALT responders vs nonresponders: −0.8 vs 0, *P* = .04
Lassailly et al^23^	France	Morbidly obese patients with biopsy-proven NASH (Lille Bariatric Cohort, 1994–2013)	Prospective observational cohort study	109	Bariatric surgery	1 y	1-y postsurgery: • NASH disappeared from 85% (95% CI 75.8–92.2) of patients • Steatosis detected in 10% of tissue (vs 60% presurgery; *P* < .0001) • Mean NAS reduced from 5 (presurgery) to 1 (*P* < .0001) • Hepatocyte ballooning and lobular inflammation reduced in 84.2% and 67.1% of samples, respectively	1-y postsurgery: • Significant improvement in fibrosis observed based on Metavir (*P* < .003) and Kleiner (*P* < .0001) scores • Fibrosis according to Metavir score was reduced in 33.8% (95% CI 23.6–45.2) of patients overall and 46.6% (95% CI 33.3–60.1) of patients with a fibrosis score ≥1 at baseline ○ Baseline score (0/1/2/3/4), n: 22/32/17/6/3 ○ 1-y, n: 35/26/11/6/2 • Fibrosis according to Kleiner score was reduced in 46.3% (95% CI 35.8–55.8) of patients overall and 51.4% (95% CI 39.3–63.4) of patients with a fibrosis score ≥1 at baseline ○ Baseline score (0/1a/1b/1c/2/3/4), n: 8/10/6/9/26/19/3 ○ 1-y, n: 26/7/4/13/15/14/2
Li et al^24^	China	Patients with pathologically diagnosed NASH	Interventional study (RCT)	78	PUFA vs saline	6 mo	Significant reduction in steatosis grade, necroinflammatory grade, and ballooning score vs control	**Significant reduction in fibrosis stage vs control:** 1.6 vs 1.1, *P* = .02
Vilar-Gomez et al^25^	Cuba	Patients with biopsy-proven NASH (single center, 2009–2013)	Prospective cohort study	293	Lifestyle	1 y	**NAS improvement ≥2 points:** 47% **NAS change from baseline:** −1.58 ± 0.27	Regression:^c^ 19% Stable: 65% Progression:^c^ 16% Change from baseline: −0.01 ± 0.02
Cusi et al^26^	USA	Patients with prediabetes or T2D and biopsy-proven NASH (NCT00994682)	Interventional study (RCT)	101	Pioglitazone vs PBO	18 mo	**>2-point reduction in NAS without worsening of fibrosis:** 58% vs 17%, *P* < .001 **Resolution of NASH:** 51% vs 19%, *P* < .001	>1-point improvement: 39% vs 25%, *P* = .130 Mean change in score: –0.5 vs 0, *P* = .039
Ratziu et al^27^	USA and Europe	Patients with NASH without cirrhosis (NCT01694849)	Interventional study (RCT)	183	Elafibranor 120 mg vs PBO	52 wk	**NASH resolved**^d^**with no worsening of fibrosis:** 19% vs 12%; OR 2.31, *P* < .045	Patients with NASH resolution after receiving elafibranor 120 mg had reduced liver fibrosis stages compared with those without NASH resolution: mean reduction of 0.65 ± 0.61 in responders vs an increase of 0.10 ± 0.98 in nonresponders; *P* < .001
Friedman et al^28^	Global	Patients with biopsy-proven NASH, NAS ≥4 with ≥1 in each component, and NASH CRN stage 1–3 liver fibrosis (CENTAUR; NCT02217475)	Interventional study (RCT)	289	Cenicriviroc^e^ vs PBO	1 y	**≥2-point NAS improvement with no worsening of fibrosis:** 15.9% vs 18.8%, *P* = .52 **Complete resolution of steatohepatitis and no worsening of fibrosis stage:** 8% vs 6%, *P* = .49	**Improvement in fibrosis by ≥1 stage with no worsening of steatohepatitis:** 20.0% vs 10.4%, *P* = .0234
Oliveira et al^29^	Brazil	Patients with biopsy-confirmed NASH	Interventional study (RCT)	53	MTF + NAC + UDCA vs MTF + UDCA vs MTF + NAC	48 wk	Statistically significant improvements from baseline in steatosis degree, ballooning, and in NAS for only the NAC + MTF group	No progression of fibrosis and inflammation in any group
Francque et al^30^	USA, Australia, Canada, Mauritius, and Europe (12 countries)	Patients with noncirrhotic, highly active NASH (NATIVE; NCT03008070)	Interventional study (RCT)	247	Lanifibranor (1200 mg or 800 mg) vs PBO	24 wk	**≥2-point SAF-A**^f^**improvement with no worsening of fibrosis:** 1200 mg: 55% vs 33%, *P* = .007 800 mg: 48% vs 33%, *P* = .07 **Resolution of NASH with no worsening of fibrosis:** 1200 mg: 49% vs 22% 800 mg: 39% vs 22% **Resolution of NASH and ≥1-stage fibrosis improvement:** 1200 mg: 35% vs 9% 800 mg: 25% vs 9%	**≥1-stage fibrosis improvement with no worsening of NASH:** 1200 mg: 48% vs 29% 800 mg: 34% vs 29%
Gurka et al^31^	USA	Patients with biopsy-confirmed NASH (PIVENS; NCT00063622)	Interventional study (RCT)	201	Vitamin E vs pioglitazone vs PBO	96 wk	Significant decrease in MetS-Z at 48 and 96 wk with pioglitazone	
Harrison et al^32^	USA	Patients with biopsy-confirmed NASH (NCT02443116)	Interventional study (RCT)	78	Aldafermin vs PBO	24 wk	**Resolution of NASH with no worsening of fibrosis:** 24% vs 9%, *P* = .20 **Resolution of NASH and ≥1-stage fibrosis improvement:** 22% vs 0%, *P* = .015 **≥2-point improvement in NAS and no worsening of fibrosis:** 62% vs 9%, *P* < .001	**≥1-stage fibrosis improvement with no worsening of NASH:** 38% vs 18%, *P* = .10
Harrison et al^33^	USA	Patients with biopsy-confirmed NASH, F1–3 fibrosis (BALANCED; NCT03976401)	Interventional study (RCT)	80	Efruxifermin (28 mg, 50 mg, or 70 mg) or PBO	16 wk	**Resolution of NASH with no worsening of fibrosis:**^g^ 28 mg, 46%; 50 mg, 54%; 70 mg, 43%; PBO, 50% **Resolution of NASH and ≥1-stage fibrosis improvement:**^g^ 28 mg, 31%; 50 mg, 39%; 70 mg, 14%; PBO, 0% **≥2-point improvement in NAS:**^g^ 28 mg, 92%; 50 mg, 77%; 70 mg, 86%; PBO, 50% **≥2-point improvement in NAS with no worsening of fibrosis:**^g^ 28 mg, 77%; 50 mg, 77%; 70 mg, 79%; PBO, 50%	**≥1-stage fibrosis improvement:**^g^ 28 mg, 54%; 50 mg, 77%; 70 mg, 36%; PBO, 0% **≥2-stage fibrosis improvement:**^g^ 28 mg, 31%; 50 mg, 39%; 70 mg, 14%; PBO, 0% **≥1-stage fibrosis improvement with no worsening of NASH:**^g^ 28 mg, 46%; 50 mg, 62%; 70 mg, 36%; PBO, 0% **≥2-stage fibrosis improvement with no worsening of NASH:**^g^ 28 mg, 23%; 50 mg, 31%; 70 mg, 14%; PBO, 0%
Huang et al^34^	Taiwan	Treatment-naive Taiwanese patients with biopsy-confirmed NASH (NCT01068444)	Interventional study (RCT)	90	Pioglitazone vs PBO	24 wk	**NASH improvement with no worsening of fibrosis:** 46.7% vs 11.1%, *P* = .002 **Resolution of NASH:** 26.7% vs 11.1%, *P* = .103 **Other:** Statistically significant improvements from baseline in steatosis degree, lobular inflammation, and NAS for pioglitazone group only	**Fibrosis:** No significant change with pioglitazone; significant increase in PBO arm (*P* = .007)
Kedarisetty et al^35^	India	Consecutive patients with biopsy-confirmed NASH (NCT01384578)	Interventional study (RCT)	69	Pentoxiphylline + vitamin E vs vitamin E alone	52 wk	No difference in reduction in NAS score between treatment groups (significant reduction in both groups, both *P* = .03)	Significantly greater reduction in fibrosis score with combination vs vitamin E alone at 1 y (*P* = .004) Significant reduction in liver stiffness by FibroScan seen in both groups (both *P* < .05)
Loomba et al^36^	USA, Canada, Australia, New Zealand, and Hong Kong	Patients with biopsy-confirmed NASH and bridging fibrosis or compensated cirrhosis (F3–F4) (ATLAS; NCT03449446)	Interventional study (RCT)	392	Selonsertib or cilofexor or firsocostat or cilofexor + selonsertib or firsocostat + selonsertib or cilofexor + firsocostat vs PBO	48 wk	**NASH resolution with no worsening of fibrosis:** F vs PBO: 2.9% vs 0%, *P* = .65 C vs PBO: 0% vs 0%, *P* = 1.00 FS vs PBO: 1.4% vs 0%, *P* = .74 CS vs PBO: 1.5% vs 0%, *P* = .76 CF vs PBO: 4.5% vs 0%, *P* = .35 **≥2-point improvement in NAS:** F vs PBO: 29% vs 11%, *P* = NS C vs PBO: 18% vs 11%, *P* = NS FS vs PBO: 14% vs 11%, *P* = NS CS vs PBO: 9% vs 11%, *P* = NS CF vs PBO: 35% vs 11%, *P* ≤ .05	**≥1-stage fibrosis improvement with no worsening of NASH:** F vs PBO: 12% vs 11%, *P* = .94 C vs PBO: 12% vs 11%, *P* = .96 FS vs PBO: 15% vs 11%, *P* = .62 CS vs PBO: 19% vs 11%, *P* = .26 CF vs PBO: 21% vs 11%, *P* = .17
Newsome et al^37^	USA, Canada, Australia, Japan, Russian Federation, and Europe (11 countries)	Patients with biopsy-confirmed NASH and BMI ≥25 kg/m^2^ ± T2D (NCT02970942)	Interventional study (RCT)	320	Semaglutide (0.4 mg, 0.2 mg, or 0.1 mg) vs PBO	72 wk	**NASH resolution with no worsening of fibrosis:** 0.4 mg: 59% vs 17%, *P* < .001 0.2 mg: 36% vs 17% 0.1 mg: 40% vs 17% **NASH resolution and improvement in fibrosis:** 0.4 mg: 37% vs 15% **≥1-point improvement in NAS:** 0.4 mg, 83%; 0.2 mg, 80%; 0.1 mg, 71%; PBO, 44%	**≥1-stage fibrosis improvement with no worsening of NASH:** 0.4 mg: 43% vs 33%, *P* = .48 0.2 mg: 32% vs 33% 0.1 mg: 49% vs 33% **≥2-stage fibrosis improvement with no worsening of NASH:** 0.4 mg, 20%; 0.2 mg, 19%; 0.1 mg, 25%; PBO, 17%
Okanoue et al^38^	Japan	Patients with biopsy-confirmed NASH	Interventional study (RCT)	48	Apararenone vs PBO	72 wk	**≥2-point improvement in NAS without worsening of fibrosis:** 20.8% vs 26.1%, *P* = .736	**≥1-stage fibrosis improvement with no worsening of NAS:** 41.7% vs 26.1%, *P* = .203 **≥1-stage fibrosis improvement:** 45.8% vs 30.4%, *P* = .291
Ratziu et al^39^	USA, Mexico, Israel, Chile, Georgia, Hong Kong, and Europe (5 countries)	Patients with biopsy-confirmed NASH, BMI ≥25–40 kg/m^2^ and T2D or prediabetes (ARREST; NCT02279524)	Interventional study (RCT)	247	Aramchol (600 mg or 400 mg) vs PBO	52 wk	**NASH resolution with no worsening of fibrosis:** 600 mg: 16.7% vs 5%, *P* = .051 400 mg: 7.5% vs 5%, *P* = .50 **≥2-point improvement in NAS**^h^**with no worsening of fibrosis** 600 mg: 25.6% vs 17.5%, *P* = .31 400 mg: 20.0% vs 17.5%, *P* = .56 **≥2-point improvement in SAF-A with no worsening of fibrosis:** 600 mg: 35.9% vs 25.0%, *P* = .16 400 mg: 25.0% vs 25.0%, *P* = .86	**≥1-stage fibrosis improvement with no worsening of NAS:** 600 mg: 29.5% vs 17.5%, *P* = .21 400 mg: 21.3% vs 17.5%, *P* = .84

Bold text denotes clinical endpoints.

ALT, alanine aminotransferase; BMI, body mass index; C, cilofexor; CF, cilofexor + firsocostat; CI, confidence interval; CRN, Clinical Research Network; CS, cilofexor + selonsertib; F, firsocostat; FS, firsocostat + selonsertib; MetS-Z, metabolic syndrome severity Z-score; MTF, metformin; NAC, N-acetylcysteine; NAFLD, nonalcoholic fatty liver disease; NAS, NAFLD Activity Score; NASH, nonalcoholic steatohepatitis; NS, not significant; OR, odds ratio; PBO, placebo; PIVENS, Pioglitazone vs Vitamin E vs Placebo for the Treatment of Non-Diabetic Patients with Nonalcoholic Steatohepatitis; PUFA, polyunsaturated fatty acid; RCT, randomized controlled trial; SAF-A, Steatosis, Activity, and Fibrosis-Activity; T2D, type 2 diabetes; UDCA, ursodeoxycholic acid.

a Improvement by ≥1 point in hepatocellular ballooning score; no increase in the fibrosis score; and either a decrease in the activity score for NAFLD to ≤3 or a ≥2-point decrease in the activity score, with a ≥1-point decrease in either the lobular inflammation or steatosis score.

b ALT response was defined as a decrease to ≤40 U/L and by ≥30% of baseline.

c Change by ≥1 point.

d Resolution of NASH as disappearance of ballooning (score 0), together with either disappearance of lobular inflammation or the persistence of mild lobular inflammation only (score 0 or 1), and resulting in an overall pathologic diagnosis of either steatosis alone or steatosis with mild inflammation.

e The cencricriviroc program has been canceled after early termination of AURORA (NCT03028740) due to lack of efficacy.

f The activity part of the SAF scoring system that incorporates scores for ballooning and inflammation.

g Only 2 patients receiving placebo were included in the biopsy-evaluable set; therefore, meaningful comparison with placebo is not possible.

h Contributed to by at least 2 of: steatosis, inflammation, ballooning.

Resolution of NASH may result in improvement in metabolic parameters. Two post-hoc analyses of the Pioglitazone vs Vitamin E vs Placebo for the Treatment of Non-Diabetic Patients with Nonalcoholic Steatohepatitis (PIVENS) study, which pooled data from patients receiving pioglitazone, vitamin E, and placebo in nondiabetic patients with NASH, found that NASH resolution was associated with favorable changes in triglycerides, high-density lipoprotein levels, and lipoprotein fractions, compared with patients without NASH resolution.^40^^,^^41^

### Risk Factors for Progression or Resolution of NASH and/or Fibrosis

Five publications reported on factors associated with regression or resolution of NASH or fibrosis (Table 3).^15^^,^^17^^,^^31^^,^^38^^,^^42^ In the PIVENS trial, factors associated with higher odds of NASH resolution included reductions in alanine aminotransferase (ALT) and aspartate aminotransferase (AST; vitamin E and pioglitazone arms), lower baseline metabolic syndrome (MetS) severity Z-score (MetS-Z; vitamin E and pioglitazone arms), and reductions in MetS-Z (placebo arm).^31^ In patients not receiving pharmacologic intervention, lower odds of NASH resolution were also reported in consistent moderate drinkers compared with consistent nondrinkers.^17^ Factors that predicted fibrosis regression included higher baseline fibrosis stage, lower baseline levels of fibrosis markers, and reduced/normal liver enzyme levels.^15^^,^^38^

**Table 3:** Predictors of NASH/Fibrosis Progression or Resolution

Reference	Country	Data source/population	Study type	N	Predictive factors
Markers of disease regression/resolution					
Vilar-Gomez et al^42^	Cuba	Patients with biopsy-proven NASH who underwent 12 mo lifestyle modification (single center, 2009–2013)	Observational cohort study	261	• Factors predicting improvement in liver fibrosis (≥1 stage) 1 y after lifestyle intervention were: ○ Age at baseline (OR 0.94; *P* = .04) ○ Normal levels of ALT at EOT (<19 U/L for women and <30 U/L for men; OR 9.7; *P* < .01) ○ Change in HbA_1c_ (OR 0.39; *P* < .01), HOMA-IR (OR 0.88; *P* = .01), platelets count (OR 1.22; *P* < .01), NFS (OR 0.27; *P* < .01) • Change in liver enzymes (ALT, AST) and other fibrosis models (APRI, FIB-4) were not associated with fibrosis improvement even after adjustments by improvements in other histologic outcomes
Ajmera et al^17^	USA	NASH CRN participants ≥21 y with biopsy-confirmed NAFLD who did not receive specific pharmacologic therapy for NASH and had alcohol use history within 2 y of initial biopsy^a^	Observational cohort study	NASH: 182 Borderline NASH: 49 NAFL: 54	• In patients with confirmed NASH, consistent modest drinkers had significantly lower odds of NASH resolution vs consistent nondrinkers (22% vs 11%; adjusted OR 0.32, 95% CI 0.11–0.92; *P* = .04)^b^^,^^c^ • Modest drinkers who reported nondrinking at follow-up had the second highest proportion with NASH resolution (17%) • Changes in lobular inflammation, hepatocyte ballooning, portal inflammation, fibrosis stage, and NAS were not significantly different between consistent drinkers and nondrinkers
Sanyal et al^15^	Global	Patients with biopsy-confirmed NASH and bridging fibrosis (F3) or compensated cirrhosis (F4)^d^	Interventional study (Phase 2b RCTs^e^)	NASH with F3 fibrosis: 217 NASH with compensated cirrhosis: 258	• In patients with bridging fibrosis at baseline, fibrosis regression was associated with: ○ Baseline Ishak fibrosis stage (4 vs 3; *P* = .040) ○ Lower baseline hepatic collagen content (*P* = .044) ○ Greater reduction in hepatic collagen over time (*P* < .0001) ○ Lower α-SMA expression at baseline (*P* = .007) ○ α-SMA reduction at wk 96 (*P* < .001) ○ Lower baseline ELF, NFS, FIB-4, and APRI (all *P* ≤ .013) ○ Smaller increase in LOXL2 over time (*P* = .001) • In patients with compensated cirrhosis at baseline, cirrhosis regression was associated with: ○ Baseline Ishak fibrosis stage (6 vs 5; *P* = .034) ○ Lower levels of α-SMA expression at baseline (*P* = .029) ○ Smaller increases in α-SMA expression or hepatic collagen content over time (both *P* < .001) ○ Lower baseline ELF, NFS, FIB-4, and APRI (all *P* ≤ .021) • Baseline levels of serum LOXL2, FibroSure/FibroTest, NAS, and severity of steatosis, lobular inflammation, and ballooning were not associated with fibrosis improvement in either group, nor were changes in serum fibrosis markers over time (apart from LOXL2 in patients with bridging fibrosis at baseline)
Gurka et al^31^	USA	Patients with biopsy-confirmed NASH (PIVENS; NCT00063622)	Interventional study (RCT)	201	• Inverse association between baseline MetS-Z and NASH resolution during treatment with pioglitazone (OR 0.38, 95% CI 0.15–0.96) and vitamin E (OR 0.41, 95% CI 0.19–0.90), but not placebo • Week 48 reductions in MetS-Z were associated with NASH resolution in the placebo group (OR 0.36, 95% CI 0.16–0.82), but not in the vitamin E and pioglitazone groups • Week 48 reductions in AST and ALT were associated with higher odds of NASH resolution in vitamin E and pioglitazone groups
Okanoue et al^38^	Japan	Patients with biopsy-confirmed NASH	Interventional study (RCT)	47	• Multivariate analysis showed that improvement in the FIB-4 index was significantly influenced by total bilirubin (*P* < .0001), GGT (*P* < .0001), ELF score (*P* < .0001), HDL-C (*P* = .0207), soluble VCAM-1 (*P* = .0215), and baseline Kleiner fibrosis staging (*P* = .0408)
Markers of disease progression and/or more advanced disease					
Sorrentino et al^58^	Italy	Obese (BMI >30 kg/m^2^) patients with NAFLD (single center, 1993–2003)	Observational cohort study	Total patients: 132 NASH: 83 Baseline fibrosis score (Brunt): 0, 85; 1, 30; 2, 15; 3, 2; 4, 0	• In the overall NAFLD population, independent predictors worsening fibrosis from baseline to follow-up were: ○ Intralobular fibronectin >1 (OR 14.1; *P* < .001) ○ Hypertension (OR 4.8; *P* = .028) ○ HOMA-IR score >10 (OR 1.9; *P* = .004) • These factors also correlated with more advanced fibrosis at baseline
Kruger et al^45^	South Africa	Patients with biopsy-proven NAFLD recruited at 3 South African centers	Observational cohort study	Total patients: 111 NASH: 41% Advanced fibrosis: 17%	• In the overall NAFLD population, APRI score increased with fibrosis stage (no/mild fibrosis: 0.7; advanced fibrosis: 1.54 • Lower NFS was also associated with more advanced fibrosis (*P* < .01), but AST:ALT ratio was not significantly associated with fibrosis stage (*P* = .06) • APRI was superior to AST:ALT ratio and comparable to NFS for predicting advanced fibrosis
Bando et al^47^	Japan	Patients with biopsy-proven NASH treated at 2 Japanese centers	Observational cohort-analytic study	36	• Serum ALT, platelet count, and hemoglobin level decreased with progression of fibrosis (*P* = .026, *P* = .001, and *P* = .001, respectively) • Histologic progression of fibrosis was also associated with increasing GA (*P* = .004) and GA/HbA_1c_ ratio (*P* = .003) • Significant inverse correlation was seen between GA/HbA_1c_ ratio with serum ALT (*r* = −0.572, *P* < .001) and platelet count (*r* = −0.663, *P* < .0001)
Jung et al^46^	South Korea	Biopsy-proven and nonbiopsy-proven NASH patients (single center, 2003–2012)	Observational cohort study	Total patients: 94 Biopsy-proven NASH: 41 Brunt fibrosis stage (biopsy-proven NASH): S1, 15; S2, 21; S3, 4; S4, 1	• In patients with biopsy-proven NASH, serum AST:ALT ratio significantly increased with fibrosis stage: ○ S1, 0.4 ± 0.2; S2, 0.6 ± 0.3; S3, 1.3 ± 0.5; S4, 2.1 (*P* = .029) • Serum HDL-C (mg/dL) significantly decreased with fibrosis stage: ○ S1, 45 ± 15; S2, 39 ± 8; S3, 38 ± 10; S4, 8 (*P* = .034) • No significant correlation was seen between fibrosis stage and BMI, triglycerides, AST, ALT, fasting blood sugar, or HOMA-IR
Cengiz et al^59^	Turkey	Consecutive patients with biopsy-proven NASH (single center, 2009–2013)	Observational cohort-analytic study	108 (F1/2: 94; F3/4: 14)	• Hepatic ACE2 immunoreactivity was seen in 65.9% of patients with mild (F1/2) fibrosis vs 28.5% of those with advanced fibrosis • Presence of ACE2 immunoreactivity was inversely correlated with fibrosis score (*r* = −0.337, *P* < .001) and was an independent predicting factor for liver fibrosis by univariate analysis (OR 0.194, 95% CI 0.042–0.897) • There were no correlations between ACE2 immunoreactivity and other histopathological features of NASH such as steatosis, inflammation, and ballooning (*P* > .05)
Huang et al^60^	Taiwan	Baseline data of RCT participants with biopsy-proven NASH (NCT01068444)	Observational cohort study	130	• There was a significant inverse correlation between fibrosis stages with: ○ Uric acid levels: F0, 7.2 mg/dL; F1, 6.5 mg/dL; F2, 6.3 mg/dL; F3/4, 6.0 mg/dL (*P* = .04) ○ Presence of hyperuricemia:^f^ F0/1, 48.4%; F2, 33.3%; F3/4, 9.1% (*P* = .01) • Multivariate logistic regression showed that decreased serum albumin (<3.5 g/dL; OR 40.0, 95% CI 4.5–300, *P* = .001) and absence of hyperuricemia (OR 5.6, 95% CI 1.5–21.7, *P* = .01) were significantly associated with advanced fibrosis
Sebastiani et al^44^	Canada	Consecutive patients with transjugular biopsy-proven NASH (single center, 2004–2013)	Observational cohort study	148	• After adjustment for age, sex, and diabetes, incidence of clinical outcomes^g^ was significantly greater (HR, 95% CI) in patients with: ○ HVPG >10 mm Hg (9.60, 3.07–30.12) ○ Histologic fibrosis stage (F3-F4; 3.14, 1.41–6.95) ○ APRI >1.5 (5.02, 1.6–15.7) ○ FIB-4 >3.25 (6.33, 1.98–20.2) ○ NFS >0.676 (11.9, 3.79–37.4) • Prognostic values of associated factors were (AUC, 95% CI): histologic fibrosis stage (0.85, 0.76–0.93), HVPG (0.81, 0.70–0.91), APRI (0.89, 0.82–0.96), FIB-4 (0.89, 0.83–0.95), NFS (0.79, 0.69–0.91), transient elastography (0.87, 0.77–0.97) • Neither histologic steatosis nor noninvasive steatosis methods predicted outcomes (AUC <0.50)
Seko et al^43^	Japan	Patients with biopsy-confirmed NASH (single center, 1999–2014)	Observational cohort study	52	• Multivariate analysis identified ALT nonresponse^h^ as a significant and independent predictor of: ○ Deterioration of NAS: HR 5.85; *P* = .031 ○ Progression of liver fibrosis: HR 4.50; *P* = .029
Sourianarayanane et al^61^	USA	Liver disease patients who underwent transjugular biopsy with pressure measurements (single center, 2001–2013)	Observational cohort study	Total patients: 142 NASH: 35 Fibrosis stage (NASH): S0, 0; S1, 9; S2, 6; S3, 8; S4, 12	• Strong correlations were reported between increasing HVPG (*r* = 0.64; *P* < .039) and WHVP (*r* = 0.63; *P* < .0001) and increasing fibrosis stage in NASH patients • Correlation was seen in all fibrosis stages, including noncirrhotic patients • At each stage of fibrosis, HVPG and WHVP measurements were lower in NASH vs HCV patients, indicating potential for underestimation of pressures or fibrosis in NASH patients
Vilar-Gomez et al^42^	Cuba	Patients with biopsy-proven NASH undertaking 12 mo lifestyle modification (single center, 2009–2013)	Observational cohort study	261	• Predictors of fibrosis progression (≥1 stage) 1 y after lifestyle intervention were: ○ High baseline BMI (OR 1.09; *P* = .01) ○ T2D at baseline (OR 2.40; *P* = .02) ○ ALT normalization (<19 U/L for women and <30 U/L for men; OR 0.21; *P* < .01) ○ Change in platelets count (OR 0.96; *P* < .01), NFS (OR 1.81; *P* < .01)
Mahamid et al^62^	Israel	Patients with biopsy-proven NASH (single center, 2015–2017)	Observational cohort study	83 Fibrosis stage: F0, 11; F1, 20; F2, 42; F3, 7; F4, 3	• Lower folate levels were significantly correlated with increasing fibrosis stage (*P* < .01). • Low B12 levels were significantly associated with a higher fibrosis grade and NASH activity (*P* < .001 and *P* < .05, respectively)
Barb et al^63^	USA	Individuals with BMI >25 kg/m^2^ recruited from the general population (single center)	Cross-sectional study	NASH: 94 NAFL or borderline NASH: 52 No NAFLD: 41	• In patients with confirmed NASH, increasing plasma FGF21 level correlated with worsening fibrosis stage (β-coefficient 83 ± 20, 95% CI 44–121, *P* < .001) • FGF21 levels were not significantly changed with worsening grades of steatosis (*P* = .6) but were higher with greater necroinflammation scores (sum of ballooning and inflammation scores; *P* = .02)
Sanyal et al^15^	Global	Patients with biopsy-confirmed NASH and bridging fibrosis (F3) or compensated cirrhosis (F4)^d^	Interventional study (Phase 2b RCTs^e^)	NASH with F3 fibrosis: 217 NASH with compensated cirrhosis: 258	• In patients with bridging fibrosis at baseline, factors associated with progression to cirrhosis included: ○ Higher baseline hepatic collagen content (*P* = .013) ○ Greater increase in hepatic collagen over time (*P* < .001) ○ Higher baseline α-SMA expression (*P* < .001) ○ Greater increase in α-SMA change at week 96 (*P* < .001) ○ Higher baseline ELF, NFS, FIB-4, FibroSure/FibroTest, and APRI (all *P* < .001), and LOXL2 (*P* = .010) ○ Greater increase in ELF (*P* < .001), APRI (*P* = .029), and LOXL2 (*P* < .001) over time ○ Lower platelets at baseline (*P* < .001) ○ Higher INR at baseline (*P* = .026) ○ Severe hepatic ballooning at baseline (grade 2 vs 0; *P* = .010) • Ishak fibrosis stage was not associated with progression to cirrhosis (*P* = .46) • In patients with compensated cirrhosis at baseline, factors associated with liver-related clinical events^i^ included: ○ Lack of improvement in Ishak fibrosis score (*P* = .027) ○ Higher baseline hepatic collagen content (*P* = .018) ○ Higher baseline ELF, NFS, FIB-4, APRI, LOXL2 (all *P* ≤ .001), and FibroSure/FibroTest (*P* = .006) ○ Higher baseline bilirubin, INR, and MELD (all *P* ≤ .007) ○ Greater increases in bilirubin, MELD, hepatic collagen, FIB-4 (all *P* ≤ .040), and LOXL2 (*P* < .001) over time ○ Lower platelets at baseline (*P* < .002) ○ Clinically significant portal hypertension at baseline (HVPG ≥10 mm Hg; *P* = .007) ○ Higher baseline HVPG and greater change over time (both *P* < .001) • Failure to achieve ≥20% decrease in HVPG, and failure to achieve HVPG <10 mm Hg and/or a ≥20% decrease (both *P* = .005)
Reddy et al^18^	USA	Electronic medical records of patients with biopsy-proven NAFLD with ≥2 biopsies ≥1 y apart (2006–2016)	Observational chart review study	NAFL: 26 NASH: 10	• In the overall population, baseline FIB-4 score, NFS, and AST:ALT ratio were all significantly higher in patients with fibrosis progression vs no progression of fibrosis, respectively: ○ FIB-4: 1.95 ± 0.96 vs 1.24 ± 0.73, *P* = .02 ○ NFS: −0.29 ± 1.24 vs −1.61 ± 1.56, *P* = .019 ○ AST:ALT ratio: 1.05 ± 0.25 vs 0.91 ± 0.72, *P* = .018 • After a median 3.8 y follow-up, FIB-4 score, NFS, and AST:ALT remained significantly higher in patients whose fibrosis had progressed • Significantly more patients with fibrosis progression had T2D at follow-up vs those without progression (30.5% vs 25.0%, *P* = .04)
Ruiz-Cassas et al^48^	USA	Patients with histologically or phenotypically confirmed NASH (GAIN study [O’Hara et al^64^] US cohort)	Retrospective cross-sectional study	NASH: 989	• By multivariate analysis,^j^ the odds of fibrosis progression were: ○ 17% higher with each year since NASH diagnosis (*P* < .001) ○ 41% lower for women than men (*P* = .016) ○ 131% higher for smokers vs nonsmokers (*P* = .004) ○ 89% higher for those with obesity (*P* = .002) ○ 395% higher when a liver transplant was proposed at diagnosis vs not proposed (*P* = .002) • Compared with those in full-time employment, the odds of fibrosis progression were: ○ 75% higher for those with part-time employment (*P* = .046) ○ 106% higher for patients who were retired (*P* = .045) ○ 108% higher for those who are unemployed (*P* = .039) ○ 2563% higher for those who are physically unable to work due to NASH or related complications (*P* = .002)^k^
Vieira Barbosa et al^65^	USA	Patients with NAFLD/NASH from the TriNetX global federated research network	Observational cohort study	NASH: 914	• Patients with baseline FIB-4 ≥2.67 experienced a substantial increase in all-cause mortality compared with those with FIB-4 <2.67 (*P* < .001)

α-SMA, alpha-smooth muscle actin; ACE2, angiotensin-converting enzyme 2; ALT, alanine aminotransferase; APRI, AST-to-platelet ratio index; AST, aspartate aminotransferase; AUC, area under the curve; BMI, body mass index; CI, confidence interval; CRN, Clinical Research Network; ELF, enhanced liver fibrosis; EOT, end of treatment; FGF21, fibroblast growth factor 21; FIB-4, fibrosis-4; GA, glycated albumin; GGT, gamma-glutamyl transferase; HbA_1c_, glycated hemoglobin; HCC, hepatocellular carcinoma; HCV, hepatitis C virus; HDL-C, high-density lipoprotein cholesterol; HOMA-IR, homeostasis model assessment of insulin resistance; HR, hazard ratio; HVPG, hepatic venous pressure gradient; INR, international normalized ratio; LOXL2, lysyl oxidase like 2; MELD, Model for End-Stage Liver Disease; MetS-Z, metabolic syndrome severity Z-score; NAFL, nonalcoholic fatty liver; NAFLD, nonalcoholic fatty liver disease; NAS, NAFLD Activity Score; NASH, nonalcoholic steatohepatitis; NFS, NASH fibrosis score; OR, odds ratio; PIVENS, Pioglitazone vs Vitamin E vs Placebo for the Treatment of Non-Diabetic Patients with Nonalcoholic Steatohepatitis; r, correlation coefficient; RCT, randomized controlled trial; T2D, type 2 diabetes; VCAM-1, vascular cell adhesion molecule-1; WHVP, wedge hepatic venous pressure.

a Participants were drawn from 3 groups: (1) the adult NAFLD Database study, (2) adults on placebo in the Pioglitazone vs Vitamin E vs Placebo for the Treatment of Non-Diabetic Patients with Nonalcoholic Steatohepatitis (PIVENS) trial, and (3) adults on placebo in the Farnesoid × Receptor Ligand Obeticholic Acid in NASH Treatment (FLINT) trial.

b Nondrinkers were defined as current “never” drinkers who had not, over the course of their lifetime, had ≥1 alcoholic drink per month during a 12-month period or had ≥3 drinks per day for ≥3 consecutive days, whereas modest drinkers were defined as those drinking ≤2 drinks on a typical day in the absence of monthly (or more frequent) heavy drinking. Consistent drinkers and nondrinkers were those whose drinking status did not change between baseline and follow-up.

c Adjusted for age at biopsy, sex, race, and ever smoker.

d Simtuzumab- and placebo-treated patients were pooled based on the assumption that simtuzumab has no biological activity, supported by its failure to improve liver histology, portal pressure, liver biochemistry, and serum fibrosis markers in RCTs.

e NCT01672866 and NCT01672879.

f Presence of hyperuricemia defined as ≥7 mg/dL for men and ≥6 mg/dL for women.

g Clinical outcomes were death, liver transplantation, and end-stage hepatic complications (HCC, ascites, spontaneous bacterial peritonitis, hepatic encephalopathy, de novo varices, or significant worsening of varices).

h ALT response was defined as a decrease of 30% or more from baseline.

i Liver-related clinical events included ascites, hepatic encephalopathy, newly diagnosed varices, esophageal variceal bleed, ≥2-point increase in Child-Pugh score and/or MELD ≥15, and death.

j The final multivariable model included age, years since diagnosis, sex, employment status, smoking status, obesity, fibrosis stage, diagnostic biopsy, vitamin E, and liver transplant proposed at diagnosis.

k Only 6 patients reported inability to work due to NASH; this OR should be interpreted with caution.

Several publications reported on factors associated with disease progression or more advanced disease; these were predominantly cross-sectional studies that evaluated markers in patients with different stages of fibrosis (Table 3). One study in Japanese patients reported that absence of ALT response to pharmacologic treatment was associated with worsening NAS or fibrosis.^43^ Two studies noted factors associated with liver-related clinical events in patients with compensated cirrhosis^15^ or NASH^44^; these included baseline (or lack of improvement in) fibrosis stage; higher baseline levels of hepatic collagen, lysyl oxidase like 2, bilirubin, international normalized ratio, and Model for End-Stage Liver Disease; lower baseline platelets; higher noninvasive tests (enhanced liver fibrosis, NAFLD fibrosis score [NFS], fibrosis-4, AST-to-platelet ratio index, FibroSure/FibroTest); and higher (or worsening/lack of improvement in) hepatic venous pressure gradient. The most prevalent factors associated with fibrosis progression or advanced fibrosis stage across the studies were higher NFS/change in NFS,^15^^,^^18^^,^^42^^,^^45^ higher AST:ALT ratio,^18^^,^^46^ ALT normalization/nonresponse,^42^^,^^43^ higher AST-to-platelet ratio index,^15^^,^^45^ higher fibrosis-4,^15^^,^^18^ lower platelets/change in platelet count,^15^^,^^42^^,^^47^ and T2D.^18^^,^^42^ The predictive value of some factors was not consistent. Serum ALT level and AST:ALT ratio, for example, were each reported in one study as not being significantly associated with patients’ fibrosis stage.^45^^,^^46^ Clinical and sociodemographic predictors of fibrosis progression included longer time since NASH diagnosis, male sex, current smoking, obesity, and lack of full-time employment.^48^

### Hepatic Complications of NASH

One study reporting data from the US National Inpatient Sample found that NASH accounted for 9.3% of all cirrhosis-related hospitalizations from 2006 to 2014, with the proportion of hospital admissions due to NASH-related cirrhosis doubling from 2006–2008 to 2012–2014.^49^

Twelve studies reported the incidence (or an increased risk) of HCC in individuals with NASH, and 6 studies reported on the prevalence of NASH in HCC (Table 4 and Table A5). A study in diabetic patients with NASH and bridging fibrosis or cryptogenic cirrhosis reported that 14.7% of the study population developed HCC over the study period (2004–2016).^50^ Studies in patients with NASH and cirrhosis reported development of HCC in 13% and 15% of patients, after a median 5.1 years and mean 6.8 years follow-up, respectively,^51^^,^^52^ and in LT wait list patients with NASH cirrhosis, the annual incidence of HCC was 2.7%.^13^ HCC prevalence in LT recipients with NASH ranged from 16.7% to 54%.^53^, ^54^, ^55^, ^56^ HCC was more common in patients with advanced (stage 3/4) fibrosis compared with early-stage (0–2) fibrosis^53^^,^^57^ and in patients with diabetes vs without.^52^ In studies that assessed the prevalence of NASH in HCC, NASH was reported in 4%–20% of HCC patients. Generally, there were no significant differences in survival outcomes between patients with NASH-HCC and non-NASH-HCC.^66^, ^67^, ^68^

**Table 4:** Incidence of HCC in Patients With NASH

Reference	Country	Data source/population	Study type	NASH participants (N)	Proportion of patients with/developing HCC
LT recipients					
Hernandez-Alejandro et al^55^	Canada	Patients undergoing LT for NASH or HCV (single center, 2000–2011)	Retrospective database review	NASH: 102	16.7% (vs 22.6% for HCV LT recipients [n = 283])
Doycheva et al^102^	USA	Patients receiving LT for HCC (UNOS database, 2002–2017)	Retrospective database review	NASH with T2D: 1193 NASH without T2D: 732	Adjusted risk of HCC, OR (95% CI): NASH with T2D vs NASH without T2D: 1.50 (1.35–1.66) NASH with T2D vs other etiologies with T2D (n = 4577): 0.60 (0.56–0.65) NASH without T2D vs other etiologies without T2D (n = 17,639): 0.49 (0.45–0.53)
Kern et al^54^	Austria	Patients undergoing primary deceased-donor LT (single center, 2002–2012)	Observational cohort study	NASH cirrhosis: 65	53.7% (vs 31.2% ALD [n = 183] and 47.7% for HCV [n = 116] LT recipients)
Zarrinpar et al^53^	USA	Patients undergoing LT for steatohepatitis (single center, 2002–2012)	Observational cohort study	NASH: 135	24%^a^ (vs 26% for ALD LT recipients [n = 182])
Jothimani et al^56^	India	Patients undergoing LT (single center, 2009–2019)	Observational cohort study	NASH (2009–2014): 85 NASH (2015–2019): 311	2009–2014: 21.2% 2015–2019: 19.9%
Fibrosis					
Vilar-Gomez et al^50^	USA	Diabetic patients with NASH and bridging fibrosis or compensated cirrhosis (single center, 2004–2016)	Observational cohort study	NASH: 191 (metformin users: 110; metformin never-users: 81)	Overall: 14.7% (over study period) (Metformin users vs never-users: 6.4% vs 25.9%) 10-y incidence: Metformin, 12%; no metformin, 40%
Hirose et al^57^	Japan	Patients with biopsy-proven NAFLD (single center, 1975–2012)	Observational cohort study	NASH: 167	n = 6^b^ HCC incidence by fibrosis stage (per 1000 person-years of follow-up): 0–2: 0.77 (95% CI 0.26–2.25) 3–4: 9.88 (95% CI 3.36–29.1)
Cirrhosis					
Amarapurkar et al^51^	India	Patients with liver cirrhosis (single center, 2010–2011)	Observational cohort study	NASH cirrhosis: 41	15% (mean follow-up 6.8 ± 1.2 y) Annual incidence: 0.46%
O'Leary et al^13^	USA	NASH/CC or HCV patients listed for an orthotopic LT (single center, 2002–2008)	Observational cohort study	NASH/CC: 217 HCV: 646	Annual incidence: 2.7% (NASH/CC patients) and 4.7% (HCV)
Vilar-Gomez et al^52^	Spain, Australia, Hong Kong, and Cuba	Patients with biopsy-proven NASH and compensated cirrhosis (multicenter, 1995–2016)	Retrospective database analysis	NASH + compensated cirrhosis: 299	n = 39 (13.0%; median follow-up 5.1 y, range 0.5–10 y) 10-y incidence with diabetes: 25% (95% CI 18–30) vs without diabetes: 7% (95% CI 3–13; *P* < .01)
Tan et al^103^	Singapore	Patients with NASH/HBV cirrhosis who were admitted to hospital for first-onset ascites (multicenter, 2004–2015)	Observational cohort study	NASH cirrhosis: 90	Baseline prevalence: 16.7%
Other					
Vieira Barbosa et al^65^	USA	Patients with NAFLD/NASH from the TriNetX global federated research network	Observational cohort study	NASH: 914	Baseline prevalence: 2.3%

ALD, alcoholic liver disease; CC, cryptogenic cirrhosis; CI, confidence interval; HBV, hepatitis B virus; HCC, hepatocellular carcinoma; HCV, hepatitis C virus; LT, liver transplantation; NAFLD, nonalcoholic fatty liver disease; NASH, nonalcoholic steatohepatitis; OR, odds ratio; T2D, type 2 diabetes; UNOS, United Network for Organ Sharing.

a All patients with NASH-HCC had advanced fibrosis (stage 3: 3%; stage 4: 97%).

b Ninety patients (40.4%) were lost to follow-up.

### Extrahepatic Comorbidities and Complications

This review identified 16 studies reporting extrahepatic complications of NASH and 21 studies reporting CV complications. Key findings are summarized below, with further details in Table 5.

**Table 5:** NASH Comorbidity Outcomes

Reference	Country	Data source/population	Study type	N	Outcomes
Colorectal cancer					
Wong et al^69^	Hong Kong	Cohort 1: Community-recruited subjects with NAFLD (n = 64) and healthy controls^a^ Cohort 2: Consecutive patients with biopsy-proven NASH from 2 hepatology clinics in Hong Kong (n = 135)	Cross-sectional study	NAFL: 199 NASH: 49 Healthy controls: 181	• In patients with biopsy-proven NAFLD, patients with NASH had a higher prevalence of adenomas (51.0% vs 25.6%; *P* = .005) and advanced neoplasms (34.7% vs 14.0%; *P* = .011) than those with NAFL • After adjusting for demographic and metabolic factors, NASH remained associated with adenomas (OR 4.89, 95% CI 2.04–11.70) and advanced neoplasms (OR 5.34, 95% CI 1.92–14.84) • Prevalence of adenomas and advanced neoplasms was similar between patients with NAFL and control subjects
Mahamid et al^71^	Israel	Patients with biopsy-proven NASH or patients without NASH who underwent screening colonoscopy (single center, 2013–2015)	Observational cohort study	NASH: 123 Non-NASH community subjects: 100	• Colonic adenomas occurred in 11% of NASH patients and 16% in the control group (*P* = .9) • NASH was significantly associated with an increased frequency of colonic hyperplastic polyps vs control group (22.7% vs 8%; *P* < .05) • Presence of NASH was associated with increased risk for colonic hyperplastic polyps (OR 1.69, *P* < .01)
Cho et al^70^	Korea	Patients who underwent screening colonoscopy within a prospective biopsy-evaluated NAFLD cohort (single center, 2013–2018)	Observational cohort study	Total patients: 476 Disease state following confirmatory liver biopsy: No NAFLD: 97 NAFLD: 194 NASH: 185	• Advanced colorectal neoplasms seen in 12% and 13% of individuals with NAFL and NASH, respectively, vs 5% of non-NAFLD patients • Low-grade tubular adenomas were seen in 50%, 35%, and 15% of NAFL, NASH, and non-NAFLD patients, respectively • NASH independently associated with a 2.8-fold increased risk for developing advanced colorectal neoplasm compared with individuals without any histologic finding of NAFLD (*P* = .049)
CKD					
Targher et al^72^	Italy	Overweight subjects with biopsy-proven NASH and nonsteatotic controls matched for age, gender, and BMI	Observational case-control study	NASH: 80 Controls: 80	• NASH patients vs control subjects had: ○ Significantly lower eGFR (75.3 vs 87.5 mL/min per 1.73 m^2^, *P* < .001) ○ Greater frequency of abnormal albuminuria (14% vs 2.5%, *P* < .01) ○ Greater frequency of CKD (25% vs 3.7%, *P* < .001) • NASH strongly associated with increased prevalence of CKD (OR 8.31, 95% CI 2.4–16.8, *P* < .001) • Histologic severity of NASH was strongly associated with either decreasing eGFR or increasing albuminuria (*P* ≤ .01), independent of other potential confounding factors
Yasui et al^74^	Japan	Patients with biopsy-proven NAFLD from 2 Japanese hospitals (2001–2009)	Cross-sectional study	Non-NASH NAFLD: 82 NASH: 92	• Prevalence of CKD was significantly higher in patients with NASH vs non-NASH NAFLD (21% vs 6%, *P* = .007) • Patients with NASH tended to have a more advanced stage of CKD than patients with non-NASH NAFLD
Fussner et al^73^	USA	Consecutive primary solitary LT recipients (single center, 1998–2004)	Observational cohort study	Total patients: 455 NASH: 47	• NASH was independently predictive of stage ≥3 CKD at 5 y post-LT (OR 2.95, *P* = .039)
Nagai et al^75^	USA	Patients with liver diseases on LT wait list (OPTN/UNOS database, 2016–2018)	Retrospective database study	Total patients: 13,016 NASH: 3848	• Presence of CKD stage 3 or 4/5 significantly increased risk of 1-y wait list mortality in NASH patients ○ Stage 3: HR 1.58, 95% CI 1.21–2.07, *P* < .001 ○ Stage 4/5: HR 1.16, 95% CI 1.03–1.31, *P* = .01
CVD					
Alkhouri et al^81^	USA	Consecutive patients undergoing liver biopsy for clinical suspicion of NAFLD (single center)	Observational cohort study	Total patients: 83 Normal: 11 NAFL: 36 NASH: 36	• Histologic severity of liver injury and inflammation strongly associated with increased cardiovascular risk and atherogenic lipid profile • Stepwise significant increase in lipid ratios with increasing histologic severity (normal biopsies vs NAFL vs NASH): ○ TG/HDL: 1.5 vs 3.2 vs 4.5 ○ TC/HDL: 3.0 vs 4.3 vs 4.8 ○ LDL/HDL: 1.6 vs 2.5 vs 2.8
Domanski et al^76^	USA	Patients with biopsy-confirmed NAFLD (single center, 2003–2009)	Observational chart review study	NASH: 219 Non-NASH NAFLD: 158	• Similar prevalence of CVD in NASH vs NAFLD (6.8% vs 6.3%) • Trend to higher incidence with NASH vs NAFLD of unstable angina (3.2% vs 1.3%), myocardial infarction (3.2% vs 1.3%), and revascularization (4.6% vs 2.5%), respectively
Imajo et al^112^	Japan	Patients with biopsy-diagnosed NAFLD from 4 Japanese medical centers	Cross-sectional study^b^	Primary cohort: • NASH: 103 • NAFL: 53 Validation cohort: • NASH: 44 • NAFL: 25	• No differences in prevalence of T2D, hypertension, dyslipidemia, and obesity between NAFL and NASH • No differences in mean serum lipid levels (TC, TG, LDL, HDL, non-HDL-C, LDL/HDL ratio) between NAFL and NASH • Significantly elevated LDL-MI in patients with NASH vs NAFL, indicating risk of CVD may be higher in patients with NASH vs NAFL or healthy subjects • Nonuse of lipid-lowering medications and NASH were independently associated with higher LDL-MI in patients with NAFLD
VanWagner et al^82^	USA	Patients who underwent first LT or simultaneous liver-kidney transplant from 2002 to 2011 (OPTN and UHC databases)	Retrospective database analysis	Total patients: 32,810 NASH as primary indication for LT: 9.7%	• NASH is an independent risk factor for MACE after LT (incidence rate ratio 1.6; 95% CI 1.1–2.24)
Patel et al^113^	USA	Patients who had coronary angiography as part of LT evaluation (single center, 2011–2014)	Observational cohort study	Total patients: 228 NASH: 53	• Significantly more patients with NASH vs HCV or alcoholic liver disease had CAD before undergoing LT (53% vs 39% vs 20%; *P* = .004) • After adjusting for age, sex, BMI, smoking, and family history, NASH was associated with a 3.12-fold increased risk of significant CAD (*P* = .005) • Other independent predictors of significant CAD were diabetes (OR 2.36; *P* = .01), dyslipidemia (OR 2.09; *P* = .04), and current or prior history of hypertension (OR 2.2; *P* = .02)
Whitsett et al^77^	USA	Patients with biopsy-proven NASH (NMEDW database, 2002–2015)	Retrospective database study	215	• Overall prevalence of AF in NASH cohort: 4.6% • Higher prevalence of AF in <65-y-old patients with NASH (4.0%) vs non-NASH/NAFLD tertiary care cohort (2.7%) and general population^c^ (2.0%) • Increased incidence of CVD in NASH patients with AF vs NASH without AF: ○ Heart failure: 54.5% vs 8.8%, *P* < .001 ○ Cerebrovascular disease: 27.3% vs 2.0%, *P* < .001 ○ Vascular disease: 54.5% vs 13.2%, *P* = .002 ○ Hypertension: 81.8% vs 43.6%, *P* = .03 ○ T2D: 81.8% vs 45.4%, *P* = .03 • NASH and AF associated with more hospital visits (4.3 vs 2.0, *P* = .006) and longer hospital stays (11.1 vs 4.5 d, *P* = .002) vs NASH alone
Lee et al^78^	Korea	Patients with T2D who underwent repeat carotid artery ultrasonography for 6–8 y and evaluations for hepatic steatosis or fibrosis at baseline (single center, 1997–2016)	Observational cohort study	Total patients: 1120 Hepatic steatosis: 636 Hepatic steatosis with significant fibrosis: 222	• Atherosclerosis progression after 6–8 y (*P* = .001 for difference): ○ No hepatic steatosis: 34.3% ○ Hepatic steatosis: 37.9% ○ Hepatic steatosis with fibrosis: 48.6% • Hepatic steatosis was significantly associated with atherosclerosis progression: adjusted OR 1.370, 95% CI 1.025–1.832 (*P* < .05) ○ Among patients with hepatic steatosis, only individuals with fibrosis showed significant association with atherosclerosis: adjusted OR 1.615, 95% CI 1.005–2.598 (*P* < .05)
Vilar-Gomez et al^52^	Spain, Australia, Hong Kong, and Cuba	Patients with biopsy-proven NASH and compensated cirrhosis (multicenter, 1995–2016)	Observational cohort study	NASH + compensated cirrhosis: 299	• Higher annualized incidence rate of total major vascular events^d^ for NASH patients with T2D (0.45, 95% CI 0.18–1.07) vs without T2D (0.28, 95% CI 0.02–1.42)
Aminian et al^114^	USA	Patients with biopsy-confirmed fibrotic NASH and obesity, without cirrhosis (single center, 2004–2016)	Retrospective cohort study	Bariatric surgery patients: 650 Medically managed patients: 508	• Higher baseline incidence of CVD in bariatric surgery candidates vs nonsurgical patients: ○ Hypertension: 83.0% vs 46.9% ○ Dyslipidemia: 73.9% vs 46.3% ○ T2D: 40.6% vs 40.6% ○ Heart failure: 6.1% vs 1.7% ○ CAD: 6.1% vs 4.2% ○ Cerebrovascular disease: 1.9% vs 1.4%
Johnson et al^79^	USA	Patients with biopsy-confirmed NAFLD with transthoracic ECG within 1 y of biopsy (single center tissue and serum repository, 2011–2016)	Retrospective cohort study	NAFL: 17 NASH: 16	• Patients with NASH had higher rates of T2D, CAD, and hypertension vs NAFL • The E/e’ ratio, a marker of left ventricular diastolic dysfunction, was significantly higher in: ○ NASH vs NAFL (*P* = .004) ○ Advanced-stage NASH vs nonadvanced NAFLD (*P* = .021) ○ High-grade NASH vs lower-grade NAFLD (*P* = .00) • The E/e’ ratio was not significantly higher with NASH vs NAFL in patients with comorbid T2D (*P* = .31)
Jothimani et al^56^	India	Patients receiving LT (single center, 2009–2019)	Retrospective observational study	Total: 1017 NASH: 396	• Strong association between CAD and NASH (HR 1.963, 95% CI 1.19–3.32; *P* = .00) • High-risk CAD^e^ was more prevalent in NASH vs non-NASH groups (7.3% vs 4.1%; *P* = .000)
Park et al^80^	South Korea	Korean subjects with biopsy-proven NAFLD	Cross-sectional study	NAFL: 216 NASH: 182	• Prevalence of NASH was greater in patients with ≥10% 10-y ASCVD risk vs <10% (50.0% vs 29.3%, *P* < .001) • Histologic severity of NAFLD predicted the 10-y ASCVD risk. Adjusted OR^f^ (95% CI) for NASH and NAFLD vs no NAFLD: ○ NASH: 4.07 (1.40–11.88) ○ NAFLD: 1.46 (0.55–3.88) ○ *P* = .014 for trend • Fibrosis severity was also a significant risk factor for ASCVD
Ruiz-Casas et al^48^	USA	Patients with histologically or phenotypically confirmed NASH (GAIN study [O’Hara et al^64^] US cohort)	Retrospective cross-sectional study	NASH: 989	• Participants with fibrosis progression appear to have higher baseline rates of CVD (6% vs 3%), hypertension (38% vs 28%), and dyslipidemia (40% vs 30%), and similar T2D (28% vs 24%) compared with those without fibrosis progression
Tan et al^103^	Singapore	Patients with NASH/HBV cirrhosis who were admitted to hospital for first-onset ascites (multicenter, 2004–2015)	Observational cohort study	NASH: 90	• CVD and metabolic conditions were more prevalent at baseline in patients with NASH cirrhosis compared with HBV cirrhosis: ○ CVD: 35.6% vs 3.6% ○ T2D: 93.3% vs 30.0% ○ Hypertension: 93.3% vs 29.1% ○ Dyslipidemia: 100% vs 9.1%
Vieira Barbosa et al^65^	USA	Patients with NAFLD/NASH from the TriNetX global federated research network	Observational cohort study	NASH: 914 NAFLD: 17,582 RISK:^g^ 62,612	• Baseline prevalence of CVD and metabolic conditions appeared generally similar in the NASH group compared with NAFLD or those at risk of NASH, respectively: ○ MetS: 13.2% vs 15.3% vs 15.0% ○ T2D: 46.2% vs 28.7% vs 35.6% ○ Hyperlipidemia: 54.7% vs 58.0% vs 66.3% ○ Hypertension: 58.8% vs 52.7% vs 57.8% ○ CVD: 68.1% vs 60.4% vs 62.5% ○ MACE: 12.9% vs 14.9% vs 17.7%

AF, atrial fibrillation; ALT, alanine aminotransferase; ASCVD, atherosclerotic cardiovascular disease; AST, aspartate aminotransferase; BMI, body mass index; CAD, coronary artery disease; CI, confidence interval; CKD, chronic kidney disease; CVD, cardiovascular disease; E/e’, ratio of early diastolic mitral inflow velocity to early diastolic mitral annulus velocity; ECG, echocardiogram; eGFR, estimated glomerular filtration rate; HBV, hepatitis B virus; HCV, hepatitis C virus; HDL, high-density lipoprotein; HDL-C, HDL cholesterol; HOMA-IR, homeostasis model assessment of insulin resistance; HR, hazard ratio; hs-CRP, high-sensitivity C-reactive protein; LDL, low-density lipoprotein; LDL-MI, LDL-migration index; LT, liver transplantation; MACE, major adverse cardiovascular events; MetS, metabolic syndrome; NAFL, nonalcoholic fatty liver; NAFLD, nonalcoholic fatty liver disease; NASH, nonalcoholic steatohepatitis; NMEDW, Northwestern Medicine Enterprise Database Warehouse; OPTN, Organ Procurement and Transplantation Network; OR, odds ratio; T2D, type 2 diabetes; TC, total cholesterol; TG, triglyceride; UHC, University HealthSystem Consortium; UNOS, United Network for Organ Sharing.

a Diagnosis based on proton magnetic resonance spectroscopy.

b NAFLD patients with dislipidemia in the primary cohort (43.4% and 58.3% of patients with NAFL and NASH, respectively) were prospectively treated in a single-arm, open-label trial, with lipid-lowering medications, including 21 patients treated with 10 mg/kg/day ezetimibe, 15 treated with 10 mg/kg/day atorvastatin, and 19 treated with 400 mg/kg/day fibrate for 12 weeks.

c Prevalence in general population determined from previously published literature.

d Major vascular events included cardiovascular, cerebrovascular, and arterial peripheral diseases.

e High-risk CAD patients underwent percutaneous balloon angioplasty along with coronary artery stent insertion before LT.

f Adjusted for age, sex, BMI, systolic and diastolic blood pressure, LDL cholesterol, TGs, AST, ALT, hs-CRP, and HOMA-IR.

g RISK includes patients with risk profile of NASH using the following formula: age >50 years, ALT >30 U/L, and (BMI >30 kg/m^2^ or T2D).

Three studies have reported an increased risk of developing colorectal cancer in patients with NASH/NAFLD (Table 5)^69^, ^70^, ^71^; these suggest that NAFLD or NASH approximately doubles the risk of developing colorectal neoplasms. Three studies reported that NASH is an independent risk factor for chronic kidney disease (CKD) or is associated with an increased risk for severe CKD post-LT (Table 5). When compared against nonsteatotic controls matched for age, gender, and body mass index (BMI), NASH was associated with an 8.3-fold increased risk of CKD.^72^ In individuals undergoing LT, the increased risk was approximately 3-fold higher in those with NASH vs those without^73^ and 3.5-fold higher in patients with NASH vs non-NASH NAFLD.^74^ The presence of severe CKD (stage 3 or 4/5) in patients with NASH was associated with significantly increased risk of 1-year LT wait list mortality.^75^

Associations between NASH and CVD were investigated in 15 studies (Table 5). Several studies reported increased risk of CV conditions with NASH compared with nonalcoholic fatty liver,^76^, ^77^, ^78^, ^79^, ^80^ and accordingly, NASH patients were found to have a more atherogenic lipid profile in NASH compared with non-NASH populations.^81^ One database study of more than 9000 patients with NASH/NAFLD reported a higher prevalence of atrial fibrillation in individuals aged <65 years with biopsy-proven NASH (4.0%) vs a non-NASH/NAFLD tertiary care cohort (2.7%) and vs a general population estimate from published literature (2.0%).^77^ Furthermore, the presence of atrial fibrillation increased the risk of other CV complications, and led to more frequent and longer hospitalizations, compared with NASH patients without atrial fibrillation.^77^ Coronary artery disease was shown to occur more frequently in patients with NASH compared with hepatitis C virus (HCV) and alcoholic liver disease, and NASH is a significant risk factor for both coronary artery disease and major adverse CV events.^82^ In addition, one study found that hepatic steatosis with fibrosis is independently associated with the progression of carotid atherosclerosis in patients with T2D.^78^

Comorbid conditions such as T2D, obesity, and hepatitis increase the risk of NASH/NAFLD and/or are associated with worse outcomes. In a single-center Belgian study, NASH was one of the most frequently reported comorbidities, occurring in 72% of participants.^83^ Another study of 1069 patients with NAFLD, 30% of whom had diabetes, showed that family history of diabetes is strongly associated with risk of NASH, fibrosis, and advanced fibrosis.^84^ T2D in NASH patients is associated with poor outcomes; in an international cohort of 299 patients with NASH, the presence of T2D was associated with a 4.59-fold increased risk of mortality, 2.46-fold increased risk of hepatic decompensation, and 4.2-fold increased risk of HCC vs patients without T2D.^52^

Obesity and overweight are established significant risk factors for NASH and fibrosis, including risk of NASH post-LT.^85^, ^86^, ^87^, ^88^, ^89^, ^90^ However, individuals with NAFLD without obesity may also be at risk of progression to NASH. One Italian study of patients with biopsy-proven NAFLD showed that increasing waist circumference was associated with increasing prevalence of severe hepatosteatosis and NASH (but not fibrosis), but in a multivariate analysis, neither NASH nor fibrosis were significantly associated with waist circumference, implying that patients with a normal waist circumference may also be at risk of NASH and fibrosis.^91^

Another study showed that the presence of NASH in patients with hepatitis was associated with significantly worse long-term outcomes, including reduced event-free survival, increased risk of HCC, HCC-free survival, transplant-free survival, and LT/death (all *P* < .01).^92^

### LT and Post-transplant Outcomes

As NASH progresses, many patients require LT, and consequently, NASH is one of the main indications for LT in many countries. The results from studies comparing the proportion of patients with different etiologies for LT suggest that in 2016, NASH accounted for ∼15%–20% of LTs in the United States and 8.4% in Europe.^93^, ^94^, ^95^, ^96^, ^97^ Both US and European studies have shown that NASH patients as a proportion of all patients undergoing LT have increased over time.^93^^,^^94^ In 2 large US studies, NASH was the second most common indication for LT.^95^^,^^97^ Additional detail on the proportion of NASH patients undergoing LT across the studies is provided in Table A6.

Studies assessing recurrence of NASH post-LT suggested that up to 50% of patients develop recurrent NASH post-LT, which progresses to fibrosis in a minority of patients.^68^^,^^98^, ^99^, ^100^, ^101^ However, very few patients (<2%) develop cirrhosis.^99^^,^^100^ Factors associated with recurrence included higher BMI (pre- and post-LT) and presence of diabetes/insulin use, metabolic syndrome, and hypertension,^98^^,^^99^^,^^101^ although one study found no association between recurrence post-LT with diabetes or hypertension.^99^

Twenty-four studies measured survival post-LT (key data presented in Table 6). Considering the large database analyses, 1-year survival was 84%–90.4% (6 studies), 3-year survival was 76.5%–85.4% (6 studies), and 5-year survival was 68.6%–84.1% (6 studies). Supporting data from smaller single-center studies are in broad agreement with the larger studies.

**Table 6:** Overall Survival in Patients With NASH Undergoing Transplantation

Reference	Country/region	Data source/population	Study type	N	Overall survival in NASH patients, %			
					1 y	3 y	5 y	10 y
Liver transplant								
Charlton et al^8^	USA	LT recipients in the USA from 2001 to 2009 (SRTR database)	Retrospective database analysis	Total patients: 35,781 NASH: 1959^a^	84	78	NR	NR
El Atrache et al^98^	USA	EMRs of hospital patients undergoing orthotopic LT for NASH or CC at Henry Ford Hospital (1996–2008)	Retrospective chart review study	Total patients: 83 NASH: 46	NR	NR	NASH + MetS: 52^b^ NASH – MetS: 90 NASH + hypertension: 61 NASH – hypertension: 86 NASH + insulin use: 58 NASH – insulin use: 88	NR
Heuer et al^115^	Germany	Patients undergoing LT due to NASH-induced cirrhosis (single center, 2007–2011)	Observational cohort study	40	NR	Overall survival at final follow-up:^c^ 65	NR	NR
Kennedy et al^116^	USA	Patients who received deceased-donor LT from 1999 to 2009 (UAB internal transplant database)	Observational chart review study	129	90	88	85	NR
Reddy et al^117^	USA	Patients undergoing curative therapy for HCC at University of Pittsburgh STI (2000–2010)	Observational cohort study	Total patients: 214 Transplanted HCC + NASH: 20	NR	Transplanted HCC + NASH: 83.3	NR	NR
VanWagner et al^118^	USA	EMRs of patients undergoing LT for NASH or AC at Northwestern Memorial Hospital and UoC (1993–2010)	Retrospective chart review study	Total patients: 242 NASH: 115	81.3	73.3	60.3	NR
Singal et al^119^	USA	Patients receiving first LT from 1994 to 2009 (UNOS database)	Retrospective database analysis	Total patients: 54,687 NASH: 1368	88.8	85.4	84.1	83.9
Wong et al^120^	USA	Patients receiving LT for HCV, NASH, ALD, and HCC from 2002 to 2012 (UNOS database)	Retrospective database analysis	Total patients: 41,289 NASH: 7100	89.3	82.5	75.5	NR
VanWagner et al^121^	USA	Patients undergoing first LT from 2002 to 2012 (OPTN database)	Retrospective database analysis	Total patients: 48,360 NASH: 5057^d^	NR	Overall survival at median 3.2 y follow-up: 73.9	NR	NR
Piazza et al^122^	USA	Patients undergoing deceased-donor LT for steatohepatitis-related cirrhosis (single center, 2005–2010)	Observational cohort study	Total patients: 143 NASH: 78	NR	NASH + CV event: 70 NASH – CV event: 90.1	NR	NR
Cholankeril et al^97^	USA	Patients undergoing LT in the USA from 2003 to 2014 (UNOS database)	Retrospective database analysis	Total patients: 63,061 NASH: 8262	89.5	83.1	77.8	NR
Thuluvath et al^123^	USA	Patients receiving first LT for NASH, CC, AC, or AIH (excluding multiorgan LT and HCC) 2002–2016 (UNOS database)	Retrospective database analysis	Total patients: 16,602 NASH: 4089	NASH cirrhosis alive: 2314 (at 2 y) NASH cirrhosis survival probability: 0.89	NASH cirrhosis alive: NR NASH cirrhosis survival probability: 0.83	NASH cirrhosis alive: 1535 (at 4 y) NASH cirrhosis survival probability: 0.77	NASH cirrhosis alive: 213 NASH cirrhosis survival probability: 0.63
Haldar et al^93^	Europe	Patients undergoing primary LT for CLD from 2002 to 2016 (ELTR database)	Retrospective database analysis	Total patients: 68,950 NASH – HCC: 1667 NASH + HCC: 1073	NASH – HCC: 84.1 NASH + HCC: 89.1	Data at 2.5 y NASH – HCC: 80.2 NASH + HCC: 76.5	NASH – HCC: 73.4 NASH + HCC: 68.6	NASH – HCC: 62.1 NASH + HCC: 46.9
Kakar et al^99^	USA	Patients undergoing LT for NASH cirrhosis (single center, 2000–2015)	Observational cohort study	226	82	NR	73	62
Kern et al^54^	Austria	Patients undergoing primary deceased-donor LT (single center, 2002–2012)	Observational cohort study	Total patients: 513 NASH cirrhosis: 65	93.2	78.5	72.1	NR
Nagai et al^124^	USA	LT patients who had NASH, HCV, or ALD from 2008 to 2017 (UNOS database)	Retrospective database analysis	Total patients: 32,660 NASH: 6344	2008–2010: 90.3 2011–2013: 90.6 2014–2015: 90.4 2016–2017: 90.4	NR	NR	NR
Tokodai et al^101^	Sweden	Patients undergoing LT for NASH or ALD (single center, 2007–2017)	Observational cohort study	Total patients: 95 NASH: 27	89	NR	NR	NR
Younossi et al^95^	USA	LT candidates with HCC listing diagnosis from 2002 to 2017 (SRTR database)	Retrospective database analysis	Total patients: 28,935 NASH + HCC: 2690 (post-transplant data available: 1631)	Post-transplant mortality, % NASH + HCC: 10.6	Post-transplant mortality, % NASH + HCC: 19.7	Post-transplant mortality, % NASH + HCC: 28.2	NR
Zarrinpar et al^53^	USA	Patients undergoing LT for steatohepatitis (single center, 2002–2012)	Observational cohort study	Total patients: 317 NASH + HCC: 33 NASH – HCC: 102	NR	NR	NASH + HCC: Overall: 62 Recurrence free: 50	NR
Holzner et al^68^	USA	Patients undergoing LT for HCC (single center, 2001–2017)	Observational cohort study	NASH-HCC: 51	NASH-HCC: 92	NASH-HCC: 86	NASH-HCC: 80	NR
Jothimani et al^56^	India	Patients undergoing LT (single center, 2009–2019)	Observational cohort study	Total patients: 1017 NASH: 396	86.6	81.8	75.9	NR
Karnam et al^125^	USA	Patients undergoing DDLT for NASH cirrhosis (SRTR database, 2002–2019)	Retrospective database analysis	6515	NR	NR	79	NR
Simultaneous liver and kidney transplant								
Singal et al^126^	USA	Simultaneous liver-kidney transplant recipients from 2002 to 2011 (UNOS database)	Retrospective database analysis	Total patients: 2606 NASH: 221	NR	NR	74	NR
Patients undergoing retransplantation								
Thuluvath et al^127^	USA	Patients receiving re-LT for NASH, CC, AC, or AHC (excluding multiorgan re-LT and HCC) from 2002 to 2016 (UNOS database)	Retrospective database analysis	Total patients: 735 NASH: 128	65	59	52	NR

AC, alcoholic cirrhosis; AHC, autoimmune hepatic cirrhosis; AIH, autoimmune hepatitis; ALD, alcoholic liver disease; CC, cryptogenic cirrhosis; CLD, chronic liver disease; CV, cardiovascular; DDLT, deceased donor liver transplant; ELTR, European Liver Transplant Registry; EMR, electronic medical record; HCC, hepatocellular carcinoma; HCV, hepatitis C virus; LT, liver transplantation; MetS, metabolic syndrome; NASH, nonalcoholic steatohepatitis; NR, not recorded; OPTN, Organ Procurement and Transplantation Network; re-LT, re-transplantation; SRTR, Scientific Registry of Transplant Recipients; STI, Thomas E. Starzl Transplantation Institute; UAB, University of Alabama at Birmingham; UNOS, United Network for Organ Sharing; UoC, University of Chicago.

a Population includes patients for whom NASH is primary (n = 1840) and secondary (n = 119) indication for LT.

b Survival data were pooled for patients with NASH (n = 46) and CC (diagnosis based on exclusion of all other forms of liver disease; n = 37).

c Final follow-up was January 2011, approximately 4 years after study initiation.

d Survival data were pooled for patients with NASH (n = 2705) and patients with probable NASH, defined as a diagnosis of CC with ≥1 component of MetS (n = 2352).

### Mortality

Key non-LT mortality data is presented in Table 7, including the Global Burden of Disease, Injuries, and Risk Factors study.

**Table 7:** NASH Mortality Outcomes in Non-LT Patients

Reference	Country	Data source/population	Study type	N	Survival outcomes
Hashizume et al^134^	Japan	Consecutive female patients with NASH or chronic HCV (single center)	Observational cohort study	Advanced fibrosis/NASH: 20 Mild fibrosis/NASH: 19	• After 85.6 mo follow-up, advanced NASH associated with shorter liver-related complication-free survival and HCC-free survival vs mild NASH, but no significant difference in overall survival
Angulo et al^104^	Multinational	Untreated patients with biopsy-proven NAFLD (multicenter, 1975–2005)	Observational cohort study	Total patients: 619 Definitive NASH: 179 Borderline NASH: 105	• Median 12.6 y follow-up • Survival free of LT was similar for definitive NASH (63.1%) and borderline NASH (60.0%), but lower than non-NASH (74.6%; *P* < .001) • In the NASH group (borderline plus definitive), survival free of LT was significantly lower in patients with fibrosis (60.2%) vs those without fibrosis (72.1%; *P* = .018)
Hirose et al^57^	Japan	Patients with biopsy-proven NAFLD (single center, 1975–2012)	Observational cohort study	Total patients: 223 NASH: 167	• Median 19.5 y follow-up • All-cause mortality was significantly higher in NASH patients than in NAFL patients (*P* = .041) • In multivariate analysis, older age (HR 1.09, 95% CI 1.05–1.14, *P* < .001) and T2D (HR 2.87, 95% CI [1.12–7.04], *P* = .021) were significantly associated with all-cause mortality
Renno et al^105^	USA	Hospitalized patients with NASH (HCUP-NIS, 2016)	Retrospective database analysis	NASH: 10,950 Matched non-NASH controls: 43,810	• Risk of in-hospital mortality was greater in NASH (3.8%) vs non-NASH patients (2%; OR 1.34, 95% CI 1.22–1.48, *P* < .0001)
Kidney failure outcomes					
Reja et al^106^	USA	Hospitalized patients with NASH and comorbid kidney failure (NIS, 2016)	Observational cohort study	NASH + kidney failure: 598 Matched controls (NASH without kidney failure): 598	• Renal failure was significantly associated with higher in-hospital mortality (3.0% vs 0.2%) ○ Adjusted OR 28.72, 95% CI 8.99–91.71, *P* < .0001
Cirrhosis outcomes					
Axley et al^49^	USA	NASH cirrhosis-related hospitalizations from US NIS (2006–2014)	Retrospective database analysis	179,104	• 3.7% inpatient mortality among hospitalizations for NASH cirrhosis
Vilar-Gomez et al^50^	USA	Diabetic patients with NASH and bridging fibrosis or compensated cirrhosis (single center, 2004–2016)	Observational cohort study	191	• Overall 2-, 4-, 6-, 8-, and 10-y cumulative mortality or liver transplant crude rates were 98%, 93%, 86%, 72%, and 52%, respectively • Cumulative 10-y transplant-free survival rate significantly lower in metformin nonusers vs users (35% vs 65%; unadjusted HR 0.42, 95% CI 0.24–0.74; *P* = .003)
GBD 2017 Cirrhosis Collaborators^107^	USA	Global Burden of Diseases, Injuries, and Risk Factors Study 2017	Retrospective database analysis	N/A	• Cirrhosis responsible for 1.32 million (2.4%) of global deaths in 2017, with NASH responsible for ∼118,000 of them
Vilar-Gomez et al^135^	USA	Patients with biopsy-proven NASH and bridging fibrosis or compensated cirrhosis (single center, 2004–2016)	Observational cohort study	236	• Vitamin E use associated with: ○ Lower overall mortality and transplant rates vs propensity score-matched nonusers (10% vs 33%, *P* < .001) ○ Higher 10-y time-dependent, adjusted, transplant-free survival rate (78% vs 49%, *P* < .01)
Vilar-Gomez et al^50^	Spain, Australia, Hong Kong, and Cuba	Patients with biopsy-proven NASH and compensated cirrhosis (multicenter, 1995–2016)	Observational cohort study	NASH + compensated cirrhosis: 299	• 87% of all deaths were liver related • 10-y survival without LT was lower in diabetic patients (38%, 95% CI 31–45) vs nondiabetic patients (81%, 95% CI 75–88) • Annualized mortality or transplant rates were 4.9 and 3.0 per 100 person-years in diabetic and nondiabetic patients, respectively (Cox-adjusted *P* < .01) • T2D increased risk of all-cause mortality or transplant 4.59-fold (95% CI 2.23–9.43) compared with no T2D • This remained significant when analyzing T2D as a time-dependent variable (adjusted HR 4.23, 95% CI 1.93–9.29, *P* < .001)
LT wait list outcomes					
Thuluvath et al^94^	USA	Patients listed for LT with primary diagnosis of NASH, CC, AC, or AIH (excluding HCC and multiple-organ transplants) from 2002 to 2016 (UNOS LT registry)	Retrospective database analysis	Total patients: 33,566 NASH: 7935	• Cumulative incidence of LT wait list death or deterioration by 3 y was 29% NASH, 28% CC and AC, 24% AIH
Kaswala et al^136^	USA	Patients listed for LT for NASH, HCV, ALD, or HCV/ALD from 2005 to 2016 (UNOS LT registry)	Retrospective database analysis	Total patients: 88,542 NASH: 22.3%	• Adjusted risk of LT wait list death lower in Hispanics and Asians vs non-Hispanic whites
Ascites-related hospitalization outcomes					
Sourianarayanane et al^137^	USA	Patients with ascites who underwent transjugular procedures (single center, 2005–2009)	Observational cohort study	Total patients: 138 NASH: 41	• NASH patients had a 1-y mortality of 38.0% • Overall unadjusted mortality was no different between NASH and non-NASH patients (48.8% vs 55.7%; *P* = .46)
Tan et al^103^	Singapore	Patients with NASH/HBV cirrhosis who were admitted to hospital for first-onset ascites (multicenter, 2004–2015)	Observational cohort study	NASH cirrhosis: 90 HBV cirrhosis: 110	• Median survival of patients with NASH cirrhosis was shorter than those with HBV cirrhosis (34.6 mo vs 72.7 mo, *P* = .001) • The 12- and 60-mo transplant-free survival was significantly shorter with NASH cirrhosis vs HBV cirrhosis ○ 12-mo: *P* = .038 ○ 60-mo: 27.2% vs 56.6%, *P* = .003 • Cumulative incidence of cirrhosis-related deaths and LT was greater for NASH cirrhosis vs HBV cirrhosis patients (65.7% vs 42.5%, *P* = .008)
HCC outcomes					
Weinmann et al^109^	Germany	Patients with NASH-associated and non-NASH-HCC (single center, 2000–2010)	Observational cohort study	Total patients: 1119 NASH-HCC: 45	• Median overall survival was 11.3 mo vs 15.5 mo for NASH-HCC and non-NASH-HCC, respectively (*P* = .287)
Kumar et al^108^	Singapore	Patients with NASH-HCC or ASH-HCC (single center HCC database, 2000–2013)	Retrospective database analysis	NASH-HCC: 54 ASH-HCC: 45	• Median overall survival was 13 ± 2.5 vs 7 ± 1.6 mo for NASH-HCC and ASH-HCC groups, respectively (*P* = .12) • Liver-related mortality was significantly better for NASH-HCC vs ASH-HCC groups (median survival 19 ± 10.3 vs 8 ± 1.3 mo, *P* = .047) • Child-Pugh score and tumor stage were significant determinants of all-cause and liver-related mortality
Young et al^66^	USA	Patients undergoing TACE for HCC (single center, 2011–2016)	Observational cohort study	Total: 220 NASH: 30	• No significant difference in overall survival in NASH-HCC vs non-NASH-HCC patients (853 vs 706 d, *P* = .48)
Billeter et al^67^	Germany	Patients operated on for noncirrhotic HCC (single center, 2001–2017)	Observational cohort study	NASH-HCC ± T2D: 34 Hepatitis-HCC ± T2D: 26 ALD-HCC ± T2D: 28	• There was no difference in overall survival among NASH-HCC (1-, 3-, and 5-y overall survival of 82.8%, 75.7%, and 71.3%), hepatitis-HCC (95.7%, 77.6%, and 60.4%), and ALD-HCC (92.0%, 79.9%, and 79.9%), *P* = .952 • NASH-HCC was associated with longer disease-specific survival than hepatitis-HCC (1-, 3-, and 5-y survival of 93%, 93%, and 87.5% vs 95.7%, 81.9%, and 63.7%, *P* = .048) • Recurrence-free survival was comparable between NASH-HCC, hepatitis-HCC, and ALD-HCC • Presence of T2D had no impact on outcomes in either liver disease
Holzner et al^68^	USA	Patients receiving LT for HCC (single center, 2001–2017)	Observational cohort study	NASH: 51 Non-NASH: 584	• There was no significant difference in overall survival between NASH-HCC and non-NASH-HCC

AC, alcoholic cirrhosis; AIH, autoimmune hepatitis; ALD, alcoholic liver disease; ASH, alcoholic steatohepatitis; CC, cryptogenic cirrhosis; CI, confidence interval; GBD, Global Burden of Disease; HBV, hepatitis B virus; HCC, hepatocellular carcinoma; HCUP, Healthcare Cost and Utilization Project; HCV, hepatitis C virus; HR, hazard ratio; LT, liver transplantation; N/A, not applicable; NAFL, nonalcoholic fatty liver; NAFLD, nonalcoholic fatty liver disease; NASH, nonalcoholic steatohepatitis; NIS, National Inpatient Sample; OR, odds ratio; T2D, type 2 diabetes; TACE, transarterial chemoembolization; UNOS, United Network for Organ Sharing.

Consolidating mortality data is challenging because of the variety of patient populations and outcomes measured; however, in general, the studies demonstrate a high mortality burden that is further exacerbated by older age or the presence of fibrosis or comorbidities such as T2D.^52^^,^^57^^,^^104^, ^105^, ^106^ Individual studies reported that NASH-related cirrhosis was responsible for ∼118,000 deaths globally in 2017,^107^ and there was a 3.7% inpatient mortality rate among patients hospitalized for cirrhosis in the United States between 2006 and 2014.^49^ There was no consistent position on the impact of NASH etiology (vs other underlying etiologies) on mortality in HCC populations.^66^^,^^67^^,^^108^^,^^109^

### Quality of Included Studies

Information on the quality assessment of included studies is provided in Tables A8 and A9.

## Discussion

The findings of this study provide a detailed picture of the course of NASH and the associated complications, as well as risk factors for the development of NASH and worsening fibrosis. Overall, 173 publications were included in this review, but considerable heterogeneity in study designs and methodologies, population sizes, study duration, outcomes, and study quality meant that direct comparison across studies was challenging.

Few studies evaluated the natural history of NASH in the absence of pharmacologic or bariatric surgery intervention, and no firm conclusions could be drawn due to study heterogeneity. Generally, findings showed that NASH progresses over time for many patients, with an increasing risk of liver-related complications such as cirrhosis and HCC with increasing fibrosis.^13^, ^14^, ^15^^,^^17^^,^^18^^,^^43^^,^^50^^,^^51^^,^^53^, ^54^, ^55^^,^^57^^,^^69^^,^^93^, ^94^, ^95^, ^96^, ^97^^,^^102^ Despite this, some studies report spontaneous NASH regression or resolution in patients not receiving pharmacologic treatment (including in placebo arms of randomized control trials), albeit in a smaller proportion than among patients receiving interventions.^20^, ^21^, ^22^^,^^24^, ^25^, ^26^, ^27^ The reasons for these spontaneous regressions are not clear, and although variance within the limitations of histologic assessments may play a role, they likely reflect behavioral changes. Interestingly, although previous studies have indicated that NASH resolution is linked to fibrosis regression,^110^^,^^111^ several interventional studies reported improvements in NASH histologic features without significant improvements in fibrosis stage.^19^, ^20^, ^21^^,^^34^^,^^37^ The reasons for this discordancy are unclear but could include differences between studies in patient baseline characteristics (eg, fibrosis stage, BMI) or study design (eg, dosing, treatment duration).^34^ It is also possible that assessment of fibrosis using continuous variables, such as the degree of liver stiffness, may have shown improvements that could not be detected using categorical outcome measures.^37^ Overall, the balance of disease trajectory data suggests that development of steatohepatitis and fibrosis are mostly reversible changes that may respond to pharmacologic interventions but also to changes in lifestyle (ie, diet and physical activity) mediated largely by weight loss. In the absence of long-term follow-up for many of the identified studies, the durability and impact of these regressions or resolutions remain unclear.

Liver biopsy remains the only validated tool for NASH diagnosis and staging, and as such, repetitive biopsies are a typical regulatory requirement for interventional studies.^128^^,^^129^ However, liver biopsies are inherently invasive, can be a burden to patients, and present logistical challenges for use in clinical trials (eg, costs, availability of sufficiently experienced pathologists).^128^^,^^129^ Therefore, development of noninvasive markers, such as blood-based biomarkers, is an active area of research,^130^^,^^131^ and indeed, both the U.S. Food and Drug Administration and the European Medicines Agency encourage their development to potentially replace liver biopsy in future clinical trials.^128^^,^^129^ The present review includes several studies that identified blood-based risk factors for NASH disease progression or regression; however, many risk factors were identified by single studies only, and the more frequently occurring factors were not always consistent across studies. Further research is needed to test and validate such biomarkers to determine their reliability and potential for use in clinical practice.^130^^,^^131^

The burden of NASH in terms of comorbidities and complications is high and growing. More than 80% of patients with NASH are estimated to have one or more comorbidity, with notably high global prevalence rates for obesity (82%), hyperlipidemia/dyslipidemia (72%), MetS (71%), hypertension (68%), and diabetes (44%).^1^ Furthermore, increasing incidence rates of NAFLD-related cirrhosis, NAFLD cirrhosis-related mortality, and NAFLD-related liver cancer have been reported.^132^ The present review confirms a high burden in terms of both hepatic complications (eg, cirrhosis and HCC) and extrahepatic manifestations (comorbid CVD and metabolic conditions; increased risk of CKD and colorectal cancer).^52^^,^^69^^,^^70^^,^^72^, ^73^, ^74^^,^^77^^,^^78^^,^^88^^,^^113^ Surprisingly, no studies were identified in this review that reported an association between NASH with other solid tumor types. This may arise from the constraints of this study’s eligibility criteria to include outcomes reported in NASH populations or subpopulations only because associations between mixed severity NAFLD populations with other extrahepatic tumor types have been reported.^7^^,^^133^ For example, a large longitudinal cohort study in patients with NAFLD, including patients with NASH and nonalcohol-related cirrhosis, has reported increased risk of uterine, stomach, and pancreatic cancers in addition to liver and colorectal cancer.^7^ Lack of universal noninvasive diagnostic methods and unreliability of liver enzymes as serum biomarkers makes it difficult to attribute cancer risk to NASH vs simple steatosis in large population-based studies,^7^ which may contribute to the limited reports identified in this NASH-specific review.

Alongside the rising prevalence of NASH and its adverse complications, the proportion of LTs resulting from NASH has increased over time in the United States, Europe, Australia, and New Zealand.^93^^,^^94^^,^^138^ Although NASH is not yet one of the most common causes of LT in Europe,^93^ it was reported to be the second most common cause for LT in 2 large US studies, behind HCV.^95^^,^^97^ The magnitude of the impact of NASH on LT in the United States may serve as an indication of the future burden in Europe, considering the projected rise in obesity across the continent.^93^ In addition, HCV is likely to decline as a cause of LT in coming years considering improvements in pharmacologic treatment options, and transplantation for NASH will therefore increase in both relative and absolute terms.^82^ Crucially, LT for NASH can be precluded by the presence of comorbidities. For example, the cause of death in individuals with NASH or cryptogenic cirrhosis is often CVD-related (whereas HCV patients are more likely to die of liver-related complications). Indicators of comorbid conditions (eg, diabetes, obesity, lipid disorders, and hypertension) should be treated aggressively to limit the risk of conditions that may be fatal or make patients ineligible for transplant.^13^

### Strengths and Limitations

This review synthesized a large body of evidence (173 publications), with most (116/151) of the quantitative studies rated as “strong” or “moderate” quality to provide a comprehensive picture of the available data.

However, we acknowledge several limitations. First, the search criteria for the present study focused on patients with NASH, and although NAFLD was included in the search strategy, publications were not included if outcome data for a NASH subgroup was absent. As NASH is the progressive phenotype of NAFLD, it is likely that some NAFLD publications lacking explicit mention of NASH were excluded. However, as all publications that described NASH in their title/abstract were included, it is unlikely that excluded publications would have focused on NASH, and they may not have presented any discrete results outside of the overall NAFLD population. Second, although the review identified an extensive body of evidence regarding the course of NASH and common complications, the evidence may not address differences in the characteristics or course of disease in different ethnic groups or different geographic regions. Furthermore, more than half of the publications reported data from the United States (88/173), and consequently, most of the evidence, especially relating to LT, is based on studies performed in the United States. Third, there was considerable heterogeneity across the included publications with regard to the sample size, study design, follow-up time, and outcomes. Furthermore, conference abstracts were excluded from any further analysis, and only full-text publications were included. Finally, despite most publications receiving “strong” or “moderate” ratings in the quality assessment, 35 publications were rated as “weak,” primarily because of missing data regarding withdrawals and dropouts, as most of these studies were observational, and inadequate controlling of confounders.

### Evidence Gaps and Recommendations

This systematic literature review included studies from 2010 to 2022, and almost half of the publications (n = 78) were published in 2019–2021. Although this shows a growing interest in research on the NASH population, data on NASH incidence and prevalence are limited and extremely variable.^2^ There is also a paucity of studies evaluating the natural history of NASH in the absence of pharmacologic or surgical intervention.

Evidence identified by this review clearly demonstrates that a significant number of interventional trials have measured histologic improvement and regression of NASH after pharmacological treatment; however, the durability and impact of regression are unclear in studies with only a few years’ follow-up. Longitudinal studies demonstrate effectively how a slowly progressing disease such as NASH occurs, but their follow-up is also not always sufficient to accurately capture NASH progression or regression. For example, Sanyal et al^15^ noted that their follow-up of 2 years is short for a slowly progressing disease such as NASH, and Ampuero et al^16^ commented that a mean follow-up time of 4.7 years may not be enough to observe a high number of prognostic outcomes (eg, HCC), whereas Hirose et al^57^ lost ∼40% of participants to follow-up over a median of 19.5 years. More longitudinal studies or real-world evidence studies over longer time frames are needed to accurately assess NASH progression/regression, especially given the expected increase in future prevalence and impact of NASH with the obesity epidemic. Harmonized study durations and patient populations and appropriate control of confounders are also needed to fully assess the prevalence and impact of NASH, which in turn could support an evidence-based focused approach to its disease burden.

Despite a wealth of studies assessing risk factors for progression of NASH and/or fibrosis, validated noninvasive biomarkers that predict patients’ disease trajectory or response to interventions are lacking. Testing and validation of such biomarkers should be prioritized, both for use in clinical practice and clinical trials, and to support our future understanding of NASH epidemiology and disease progression. Future studies should also focus on improving our understanding of NASH progression and its association with comorbidities, morbidity, and mortality, as well as associated risk factors. Except for colorectal cancer, there are limited data on the increased risk of solid tumors specifically in NASH populations. It would be beneficial for studies assessing the risk of solid tumors to stratify patients by the degree of steatohepatitis to determine if increased risks are incurred once patients’ disease progresses from nonalcoholic fatty liver to NASH.

## Conclusions

NASH is associated with significant morbidity and mortality, an increased risk of comorbidities that include HCC, CKD, and CVD, and imposes an increasing burden among LT patients. Although it is difficult to predict, NASH/NAFLD prevalence is expected to increase in parallel with the obesity and diabetes epidemics, exacerbated by a lack of general awareness, pharmacologic treatments, or a clear care pathway. Longitudinal studies with longer follow-up are required to truly understand the impact of NASH on patient outcomes.
