# Supplementary material for: The Burden of Nonalcoholic Steatohepatitis: A Systematic Review of Epidemiology Studies
Source: Gastro Hep Adv. 2022 Jul 19;1(6):1049–87. doi: 10.1016/j.gastha.2022.06.016 (PMC11307414; doi:10.1016/j.gastha.2022.06.016)
Supplement: Supplementary Materials [file mmc1.docx]

Contents

[Supplementary Results 2](#_Toc74308041)

[Study and patient characteristics 2](#_Toc74308042)

[Quality of included studies 3](#_Toc74308043)

[Supplementary Figure 4](#_Toc74308044)

[Supplementary Tables 5](#_Toc74308045)

# Supplementary Results

## Study and patient characteristics

Across the identified studies, epidemiologic data associated with NASH were derived from primary data collection and various secondary sources including electronic medical records, regional/national healthcare system registries, disease-specific registries, and university hospital medical chart data. Most publications describe observational studies, including 85 cohort studies, four cohort-analytic studies, 18 cross-sectional studies, six case-control studies, seven chart reviews, and 31 retrospective database analyses. There were 22 randomized controlled trial publications.

Most publications describe studies based in North America (52.0%), followed by Eastern Asia (15.0%), and Europe (13.3%). Multinational data was reported in 15 publications (8.7%). Key outcomes captured across the studies included disease progression or regression, complications, risk factors, and mortality (**Supplementary Table 7)**. Forty-six publications described patient populations with NASH and co-morbid disease, including obesity or morbid obesity, T2D, chronic kidney disease (CKD), dyslipidemia, and metabolic syndrome. A further 113 publications reported the prevalence of comorbid disease in NASH patients in their baseline demographic data, demonstrating an overall high prevalence of comorbid disease in this population.

Methodologies for NASH diagnosis and assessment of patients’ fibrotic severity were heterogeneous, with the most common being liver biopsy (n = 108), followed by ultrasonographic-guided liver biopsy (n = 4), ultrasonography alone (n = 3), and presence of metabolic syndrome (n = 4). Other techniques included histopathologic assessment and clinical history, and liver biopsy and/or imaging. The method of diagnosis or assessment was not reported in 30 studies, likely due to the retrospective nature of study designs that analyzed secondary data sources.

## Quality of included studies

The 151 quantitative studies were assessed using the EPHPP tool (<https://www.ephpp.ca/quality-assessment-tool-for-quantitative-studies/>), of which 44 studies were rated 'Strong', 72 were rated 'Moderate' and 35 were rated as 'Weak' (**Supplementary Table 8**). Inadequate control of confounders was a key feature of many studies, noted for most publications with ‘Weak’ or ‘Moderate’ global ratings. Missing data regarding withdrawals and study dropouts was another key reason for a global rating of ‘Weak’ since most of the studies were observational.

Quality of the 22 included randomized controlled trial publications was mixed; quality assessment is shown in **Supplementary Table 9**. Fifteen studies scored well on five or more of the seven categories (Cusi 2016; Friedman 2018; Ratziu 2016, Hoofnagle 2013; Promrat 2010; Sanyal 2010; Harrison 2021a; Harrison 2021b; Huang 2021; Okanoue 2021; Newsome 2021; Ratziu 2021; Kedarisetty 2021; Francque 2021; Loomba 2021). One study scored poorly in three areas (inadequate concealment of treatment allocation, no blinding, and unexpected imbalances in dropouts between groups; de Oliveira 2019), one did not carry out randomization appropriately (Sanyal 2019), and another scored poorly on treatment blinding (Li 2015). Four studies did not provide a clear enough description to assess quality in four or more categories (Corey 2015; Corey 2019; Younossi 2018; Gurka 2021).

# Supplementary Figure

**Figure A1.** Flow diagram of publications included and excluded from systematic review

*
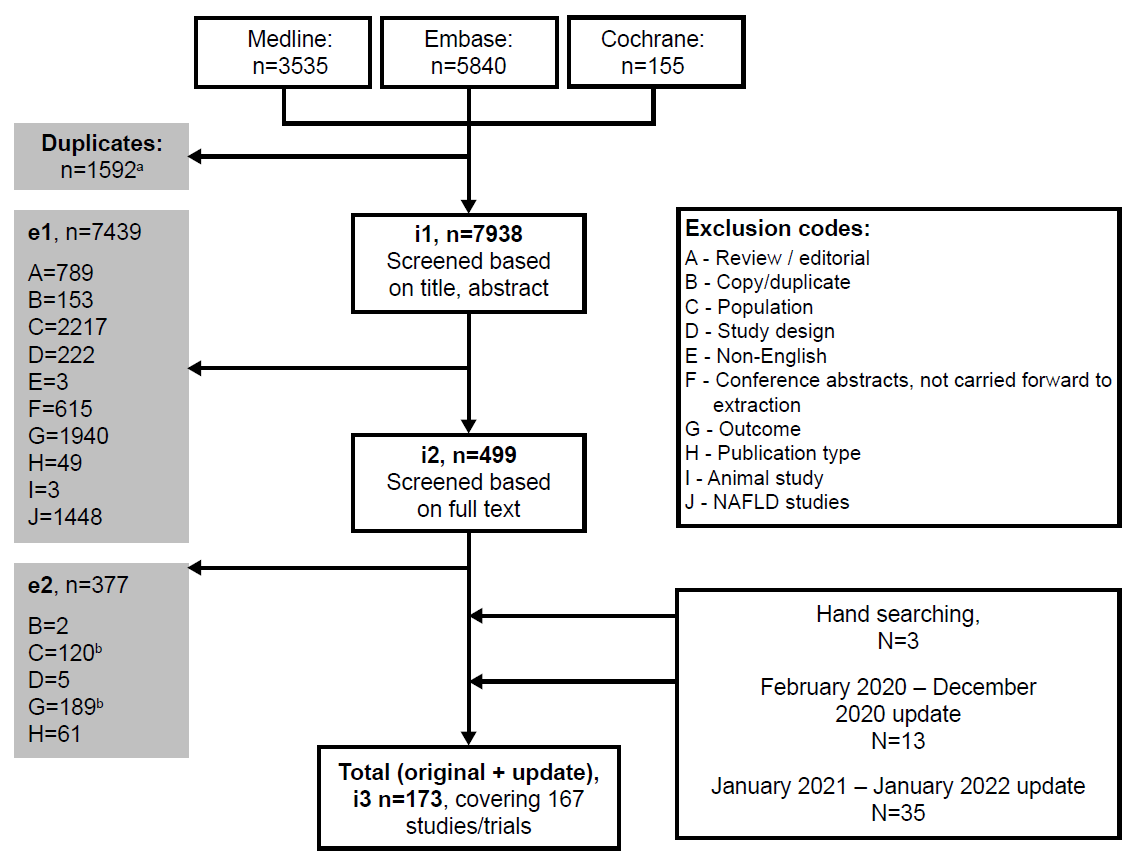
*

e1, excluded studies after title/abstract screening stage; e2, excluded studies after full-text review stage; i1, studies to screen at title/abstract stage; i2, studies to screen at full-text review stage; i3, total included publications (and studies) after full-text review stage for original report and 2021 search update; NAFLD, non-alcoholic fatty liver disease.

*^a^*De-duplication: Endnote, n=1352; DRG database, n=240; *^b^*A single publication each was not accessible.

# Supplementary Tables

**Table A1.** Search strategies for identification of publications for systematic review

| **#** | **Searches - Embase <1974 to 2022 Week 2>*^a^*** | **Results** | | |
| --- | --- | --- | --- | --- |
|  |  | **Primary SLR** | **Update 1** | **Update 2** |
| 1 | exp nonalcoholic fatty liver/ | 38036 | 46829 | 54019 |
| 2 | (non-alcoholic steatohepatitis or nonalcoholic steatohepatitis or NASH or NAFLD).mp. | 37523 | 44179 | 50752 |
| 3 | exp nonalcoholic steatohepatitis/ | 38036 | 4749 | 7018 |
| 4 | 1 or 2 or 3 | 48053 | 55905 | 64195 |
| 5 | exp risk factor/ | 1002740 | 1084643 | 1181833 |
| 6 | exp disease course/ | 3283204 | 3482544 | 3758909 |
| 7 | exp comorbidity/ | 254458 | 285139 | 323052 |
| 8 | exp complication/ | 1099089 | 1191683 | 1307309 |
| 9 | or/5-8 | 4943489 | 5273370 | 5705008 |
| 10 | 4 and 9 | 15322 | 17879 | 20676 |
| 11 | exp animal/ not (exp human/ and exp animal/) | 4574286 | 4367840 | 4504220 |
| 12 | exp note/ or exp editorial/ or exp letter/ or exp case report/ or exp Case study/ or exp Abstract report/ or (review or letter or editorial or note).pt. | 7315375 | 7555031 | 7946002 |
| 13 | or/11-12 | 11651963 | 11696067 | 12216522 |
| 14 | 10 not 13 | 9478 | 11155 | 12847 |
| 15 | limit 14 to conference abstracts | 4674 | – | – |
| 16 | 14 not 15 | 4804 | – | – |
| 17 | limit 16 to English language | 4445 | – | – |
| 18 | limit 17 to yr="2010 -Current" | 3782 | – | – |
| 19 | limit 15 to yr="2017 -Current" | 1996 | – | – |
| 20 | or/18-19 | 5778 | – | – |
| 21 | limit 14 to English language | – | 10784 | 12449 |
| 22 | limit 21 to conference abstracts | – | 5584 | 6199 |
| 23 | 21 not 22 | – | 5200 | 6250 |
| 24 | (Feb* 2020 or Mar* 2020 or Apr* 2020 or May* 2020 or Jun* 2020 or Jul* 2020 or Aug* 2020 or Sep* 2020 or Oct* 2020 or Nov* 2020 or Dec* 2020).dp. | – | 445544 | – |
| 25 | 22 and 24 | – | 507 | – |
| 26 | 23 and 24 | – | 241 | – |
| 27 | limit 22 to yr="2021 -Current" | – | 0 | – |
| 28 | limit 23 to yr="2021 -Current" | – | 18 | – |
| 29 | 25 or 26 or 27 or 28 | – | 766 | – |
| 30 | (Jan* 2021 or Feb* 2021 or Mar* 2021 or Apr* 2021 or May* 2021 or Jun* 2021 or Jul* 2021 or Aug* 2021 or Sep* 2021 or Oct* 2021 or Nov* 2021 or Dec* 2021 or Jan* 2022).dp. | – | – | 821857 |
| 31 | 22 and 30 | – | – | 466 |
| 32 | 23 and 30 | – | – | 568 |
| 33 | limit 22 to yr="2021 -Current" | – | – | 481 |
| 34 | limit 23 to yr="2021 -Current" | – | – | 1130 |
| 35 | 31 or 32 or 33 or 34 | – | – | 1611 |
| **#** | **Searches - Ovid MEDLINE(R) and Epub Ahead of Print, In-Process & Other Non-Indexed Citations and Daily <1946 to January 17, 2022>*^a^*** | **Results** | | |
|  |  | **Primary SLR** | **Update 1** | **Update 2** |
| 1 | exp nonalcoholic fatty liver/ | 11275 | 13331 | 17306 |
| 2 | (non-alcoholic steatohepatitis or nonalcoholic steatohepatitis or NASH or NAFLD).mp. | 20552 | 23950 | 27226 |
| 3 | exp nonalcoholic steatohepatitis/ | 11275 | 13331 | 17306 |
| 4 | 1 or 2 or 3 | 22914 | 26707 | 30581 |
| 5 | exp risk factor/ | 803918 | 846970 | 907506 |
| 6 | exp disease progression/ | 174191 | 185340 | 198803 |
| 7 | exp comorbidity/ | 105132 | 112852 | 121781 |
| 8 | complication*.mp. | 3006217 | 3127741 | 3268299 |
| 9 | or/5-8 | 3743770 | 3906909 | 4105253 |
| 10 | 4 and 9 | 7922 | 8960 | 10715 |
| 11 | exp animal/ not (exp human/ and exp animal/) | 4672546 | 4772383 | 4939350 |
| 12 | exp note/ or exp editorial/ or exp letter/ or exp case report/ or exp case study/ or (review or letter or editorial or note).pt. | 5910216 | 6190814 | 6471348 |
| 13 | or/11-12 | 10354851 | 10730013 | 11168315 |
| 14 | 10 not 13 | 4573 | 5211 | 6329 |
| 15 | limit 14 to English language | 4212 | 4829 | 5933 |
| 16 | limit 15 to yr="2010 -Current" | 3541 | – | – |
| 17 | limit 15 to dt=20200201-20201231 | – | 241 | – |
| 18 | limit 15 to yr="2021 -Current" | – | 3 | – |
| 19 | or/17-18 | – | 241 | – |
| 20 | limit 15 to dt=20201231-20220112 | – | – | 534 |
| 21 | limit 15 to yr="2021 -Current" | – | – | 706 |
| 22 | or/20-21 | – | – | 723 |
| **#** | **Cochrane Central Register of Controlled Trials January 17, 2022; Cochrane Database of Systematic Reviews 2005 to January 17, 2022; Database of Abstracts of Reviews of Effects 1st Quarter 2016; Health Technology Assessment 4th Quarter 2016; NHS Economic Evaluation Database 1st Quarter 2016*^a^*** | **Results** | | |
|  |  | **Primary SLR** | **Update 1** | **Update 2** |
| 1 | exp nonalcoholic fatty liver/ | 0 | 0 | – |
| 2 | (non-alcoholic steatohepatitis or nonalcoholic steatohepatitis or NASH or NAFLD).mp. | 2620 | 3003 | 3454 |
| 3 | exp nonalcoholic steatohepatitis/ | 0 | 0 | – |
| 4 | 1 or 2 or 3 | 2620 | 3003 | – |
| 5 | exp risk factor/ | 25631 | 25532 | 26467 |
| 6 | exp Disease Progression/ | 7238 | 7327 | 7691 |
| 7 | exp comorbidity/ | 3713 | 3731 | 3865 |
| 8 | complication*.mp. | 150836 | 152396 | 157997 |
| 9 | or/5-8 | 180887 | 182539 | – |
| 10 | 4 and 9 | 218 | 247 | – |
| 11 | exp animal/ not (exp human/ and exp animal/) | 46 | 12 | 12 |
| 12 | exp note/ or exp editorial/ or exp letter/ or exp case report/ or exp case study/ or (review or letter or editorial or note).pt. | 10978 | 11278 | – |
| 13 | or/11-12 | 11024 | 11290 | – |
| 14 | 10 not 13 | 218 | 246 | – |
| 15 | limit 14 to English language | 177 | – | – |
| 16 | limit 15 to yr="2010 -Current" | 155 | – | – |
| 17 | limit 14 to English language [Limit not valid in Cochrane Database of Systematic Reviews; records were retained] | – | 195 | – |
| 18 | limit 15 to yr="2020 -Current" | – | 13 | – |
| 19 | 5 or 6 or 7 or 8 | – | – | 189375 |
| 20 | 2 and (Risk Factors or Disease Progression or comorbidity or complication*).mp. | – | – | 523 |
| 21 | exp editorial/ or exp letter/ or exp Case Reports/ or exp Case Reports/ or (review or letter or editorial or note).pt. | – | – | 11575 |
| 22 | 11 or 21 | – | – | 11587 |
| 23 | 2 not 22 | – | – | 3446 |
| 24 | limit 23 to english language | – | – | 2223 |
| 25 | limit 24 to yr="2021 -Current" | – | – | 241 |

SLR, systematic literature review.

*^a^*Searches included terms for free text or Medical Subject Heading (MeSH) for Medline and the Cochrane Library, or Emtree terms for EMBASE, related to non-alcoholic steatohepatitis (NASH) or non-alcoholic fatty liver disease (NAFLD), and combined with terms related to outcomes of interest.

**Table A2.** Hand-searched conference proceedings and Health Technology Assessment agencies screened for systematic review

| **Conference proceedings** | **Health Technology Assessment agencies** |
| --- | --- |
| European Association for the Study of Diabetes | UK National Institute for Health and Care Excellence |
| American Diabetes Association Scientific Sessions | Scottish Medicine Consortium |
| American Association for the Study of Liver Diseases | Canadian Agency for Drugs and Technologies in Health |
| International Liver Congress | Pharmaceutical Benefits Advisory Committee |
| European Association for the Study of Liver Diseases | French Haute Autorité de Santé |
| Asian Pacific Association for the Study of the Liver |  |
| International Society for Pharmacoeconomics and Outcomes Research |  |

**Table A3.** Criteria for eligibility to systematic review

| Criteria | Include | Exclude |
| --- | --- | --- |
| Population | - Patients with NASH - Patients with NAFLD having subgroup data separately for NASH | - Patients without NASH or NAFLD |
| Interventions / Comparators | - No restriction | - No restriction |
| Outcomes | Epidemiologic outcomes/measures on:   - Disease progression (associations between risk factors/surrogate endpoints/co-morbidities and disease progression), including:   - Markers/predictors of progression (inflammation/liver/lipid and metabolic/histological biomarkers)   - Disease progression rates - Risk factors/surrogate endpoints/co-morbidities linked/associated with the development of NASH - Clinical outcomes, including:   - Cardiovascular outcomes   - Mortality - Complications, including but not limiting to:   - Hepatocellular carcinoma   - Liver transplant,   - Cirrhosis   - Esophageal variceal bleeding   - Ascites   - Hepatic encephalopathy - Disease reversibility or resolution of NASH | - Other outcomes |
| Study design/setting | - No restriction | - Case studies, and case-control studies, editorials, letters, commentaries - Reviews - Systematic reviews*^a^* |
| Language of publication | - English-language publications | - Studies published in languages other than English |
| Date of publication | - Full-text publications: 2010−January 2022 - Conference abstracts: 2017−2022*^b^* | - Full-text publications: published before 2010 |

NAFLD, non-alcoholic fatty liver disease; NASH, non-alcoholic steatohepatitis.

*^a^*The reference lists of systematic literature reviews were included in the supplementary searches.

*^b^*After an amendment to the protocol, conference abstracts were included but not extracted for analysis. During title/abstract screening, conference abstracts identified from the electronic database with NAFLD data were also considered prior to the decision to focus on NASH.

**Table A4.** Publications meeting eligibility criteria for systematic review (173 publications representing 167 studies)

| Publication | Country | Publication | Country*^a^* |
| --- | --- | --- | --- |
| *Multinational studies (n=15)* | | | |
| Angulo 2015 | USA, Iceland, Denmark, Scotland, Australia and Thailand | Choi 2020 | Canada and  The Netherlands |
| Francque 2021 | USA, Australia, Canada, Mauritius and Europe (12 countries) | Friedman 2018 | Global |
| Haldar 2019 | Europe  (33 countries) | Loomba 2021 | USA, Canada, Australia, New Zealand and Hong Kong |
| Newsome 2021 | USA, Canada, Australia, Japan, Russian Federation and Europe (11 countries) | Pelusi 2019 | Italy and Finland |
| Pons 2021 | France, Romania, Spain, Canada, Austria, Switzerland and the UK | Ratziu 2016 | Europe and the USA |
| Ratziu 2021 | USA, Mexico, Israel, France, Germany, Italy, Chile, Lithuania, Georgia, Romania and Hong Kong | Safar Zadeh 2013 | Global |
| Sanyal 2019 | Global | Vilar-Gomez 2021 | Spain, Australia, Hong Kong and Cuba |
| Younossi 2018 | Global |  |  |
| *North American studies (n=89)* | | | |
| Ajmera 2018 | USA | Alkhouri 2010 | USA |
| Alkhouri 2012 | USA | Aminian 2021 | USA |
| Asfari 2017 | USA | Axley 2019 | USA |
| Bambha 2012 | USA | Barb 2019 | USA |
| Bril 2014 | USA | Charlton 2011 | USA |
| Cholankeril 2017 | USA | Corey 2019*^b^* | USA |
| Corey 2015*^b^* | USA | Cusi 2016 | USA |
| Domanski 2012 | USA | Doycheva 2019 | USA |
| El Atrache 2012 | USA | Ferslew 2015 | USA |
| Fussner 2014 | USA | Ghoneim 2020 | USA |
| Gill 2011 | USA | Golabi 2018 | USA |
| Gurka 2021*^b^* | USA | Harrison 2021a | USA |
| Harrison 2021b | USA | Henson 2020 | USA |
| Hernandez-Alejandro 2012 | Canada | Hindi 2013 | USA |
| Holzner 2021 | USA | Hoofnagle 2013*^b^* | USA |
| Johnson 2021 | USA | Kakar 2019 | USA |
| Karnam 2022 | USA*^c^* | Kaswala 2020 | USA |
| Kennedy 2012 | USA | Kwong 2020 | USA |
| Loomba 2012 | USA | Mazumder 2020 | USA |
| Molnar 2019 | USA | Nagai 2019 | USA |
| Nagai 2021 | USA | O'Leary 2011 | USA |
| Pagadala 2012 | USA | Park 2011 | USA |
| Patel 2018a | USA | Patel 2018b | USA |
| Patel 2021 | USA | Piazza 2016 | USA |
| Promrat 2010 | USA | Reddy 2012 | USA |
| Reddy 2020 | USA | Reha 2014 | USA |
| Reja 2021 | USA | Renno 2021 | USA |
| Rivas 2021 | USA | Ruiz-Casas 2021 | USA*^d^* |
| Sanyal 2010*^b^* | USA | Satapathy 2017 | USA |
| GBD 2020 | USA | Sebastiani 2015 | Canada |
| Sheikh 2012 | USA | Singal 2013 | USA |
| Singal 2014 | USA | Singal 2016 | USA |
| Sourianarayanane 2013 | USA | Sourianarayanane 2017a | USA |
| Sourianarayanane 2017b | USA | Stine 2015 | USA |
| Stine 2018 | USA | Thuluvath 2018a | USA |
| Thuluvath 2018b | USA | Thuluvath 2019b | USA |
| Thuluvath 2019a | USA | Tseng 2021 | USA |
| Udelsman 2019 | USA | Ulitsky 2010 | USA |
| VanWagner 2012 | USA | VanWagner 2015 | USA |
| VanWagner 2016 | USA | Vieira Barbosa 2021 | USA*^e^* |
| Vilar-Gomez 2019 | USA | Vilar-Gomez 2020 | USA |
| Weingarten 2011 | USA | Whitsett 2019 | USA |
| Wong 2014a | USA | Wong 2014b | USA |
| Young 2020 | USA | Younossi 2019 | USA |
| Zarrinpar 2019 | USA |  | USA |
| *European studies (n=23)* | | | |
| Ampuero 2021 | Spain | Anty 2016 | France |
| Auguet 2014 | Spain | Bechmann 2010 | Germany |
| Billeter 2021 | Germany | D’Ambrosio 2021 | Italy |
| Denkmayr 2018 | Austria | Fierbinteanu-Braticevici 2011 | Romania |
| Fracanzani 2011 | Italy | Hermans 2018 | Belgium |
| Heuer 2012 | Germany | Kern 2019 | Austria |
| Lassailly 2015 | France | Marti-Aguado 2021 | Spain |
| Mikolasevic 2021 | Croatia | Nilsson 2019 | Sweden |
| Pirvulescu 2012 | Romania | Russo 2021 | Italy |
| Sorrentino 2010 | Italy | Targher 2010 | Italy |
| Tokodai 2019 | Sweden | Weinmann 2015 | Germany |
| van den Berg 2018 | The Netherlands |  |  |
| *Rest of the World studies (n=46)* | | | |
| Al-Hamoudi 2020 | Saudi Arabia | Amarapurkar 2013 | India |
| Bando 2012 | Japan | Cengiz 2015 | Turkey |
| Chen 2019 | China | Chisholm 2012 | Australia |
| Cho 2019 | South Korea | de Oliveira 2019 | Brazil |
| Eshraghian 2019 | Iran | Eshraghian 2020 | Iran |
| Hashizume 2013 | Japan | Hirose 2020 | Japan |
| Huang 2015*^f^* | Taiwan | Huang 2021*^f^* | Taiwan |
| Imajo 2014 | Japan | Jung 2014 | South Korea |
| Kawanaka 2019 | Japan | Kedarisetty 2021*^g^* | India |
| Kim 2015 | South Korea | Kimura 2010 | Japan |
| Koo 2017 | South Korea | Kruger 2011 | South Africa |
| Kumar 2020 | Singapore | Jothimani 2021 | India |
| Lee 2020a | Korea | Lee 2020b | Korea |
| Li 2015 | China | Ma 2021 | China |
| Mahamid 2017 | Israel | Mahamid 2018 | Israel |
| Michitaka 2010 | Japan | Nah 2021 | South Korea |
| Okanoue 2021 | Japan | Oliveira 2019 | Brazil |
| Park 2021 | South Korea | Seko 2015 | Japan |
| Shima 2013 | Japan | Singh 2010 | India |
| Tan 2021 | Singapore | Tanaka 2011 | Japan |
| Vilar-Gomez 2015*^h^* | Cuba | Vilar-Gomez 2017*^h^* | Cuba |
| Wang 2018 | China | Wong 2011 | China (Hong Kong) |
| Yang 2021 | China | Yasui 2011 | Japan |

*^a^*Country refers to the origin of the data analyzed.

*^b^*Linked publications (randomized controlled study that compared pioglitazone, vitamin E and placebo in patients with non-alcoholic steatohepatitis without diabetes [NCT00063622]).

*^c^*Karnam 2022 was published in Canada and supported by Toronto general hospital, but reported data from US-based Scientific Registry of Transplant Recipients.

*^d^*Ruiz-Casas 2021 reported data from the US cohort of the multinational Global Assessment of the Impact of NASH (GAIN) study.

^e^Vieira Barbosa 2021 reported US data from the TriNetX global federated research network.

^f^Linked publications (randomized controlled study that investigated the safety and efficacy of pioglitazone in patients with non-alcoholic steatohepatitis (NCT01068444).
^g^This study (NCT01384578) has been withdrawn due to lack of funds.

*^h^*Linked publications (prospective study of patients with histologically proven non-alcoholic steatohepatitis undergoing lifestyle intervention).

**Table A5.** Prevalence of NASH in patients with HCC

| Publication | Country | Data source/ population | Study type | N | % having NASH | Outcomes |
| --- | --- | --- | --- | --- | --- | --- |
| Weinmann 2015 | Germany | Patients with NASH-associated and non-NASH HCC (Single-center,  2000–2010) | Observational cohort study | 1119  NASH: 45 | 4% | Shorter median overall survival in NASH-HCC vs non-NASH HCC   - 11.3 vs 15.5 months (*P* = .287) |
| Younossi 2019 | USA | LT candidates with HCC listing diagnosis from 2002 –2017  (SRTR database) | Retrospective database analysis | 28,935  NASH: 2690 | 2.1% in 2002, 16.2% in 2016 (7.7-fold increase)  Prevalence of HCC in NASH-LT candidates increased 11.8-fold | - Post-LT recurrence of HCC in NASH patients, 6.3%*^a^* - 1-year mortality, 10.6% - 5-year mortality, 28.2% |
| Young 2020 | USA | Patients undergoing TACE for HCC  (Single-center, 2011–2016) | Observational cohort study | 220  NASH: 30 | 14% | No significant difference in time to progression, overall survival, or number of complications in NASH-HCC vs non-NASH-HCC patients |
| Billeter 2021 | Germany | Patients with biopsy-confirmed non-cirrhotic HCC, with or without T2D | Retrospective database analysis | NASH-HCC ± T2D: 34  Hepatitis-HCC ± T2D: 26  Alcohol-related HCC ± T2D: 28 | 17%^b^ | - No significant difference in overall survival and recurrence-free survival between NASH-HCC, hepatitis-HCC, and alcoholic-HCC - Longer disease-specific survival with NASH-HCC versus hepatitis-HCC - T2D had no impact on oncologic outcomes in either liver disease. |
| Holzner 2021 | USA | Patients receiving  LT for HCC  (Single-center, 2001–2017) | Retrospective study | NASH: 51  Non-NASH: 584 | Receiving LT:  2001–2008: 4.4% 2017: 15.6% | No significant difference in overall survival between NASH-HCC and non-NASH-HCC |
| Jothimani 2021 | India | Patients receiving  LT, including those with HCC  (Single-center, 2009–2019) | Retrospective observational study | Total HCC: 199  NASH: 80 | NASH+HCC:  2009–2014: 21.2%  2015–2019: 19.9% | No data |

LT, liver transplantion; HCC, hepatocellular carcinoma; NASH, non-alcoholic steatohepatitis; SRTR, Scientific Registry of Transplant Recipients; T2D, type 2 diabetes; TACE, transcatheter arterial lipiodol chemoembolization; USA, United States of America.

*^a^*Post-transplant data were available for 1631 of 2690 patients with NASH-HCC.

*^b^*Calculated based on proportion of total HCC patients with NASH-related HCC (N=62/365).

**Table A6.** Proportion of patients undergoing liver transplant with NASH

| Publication | Country/ region | Data source | Study type | Patients | N | Proportion of patients receiving LT who have NASH |
| --- | --- | --- | --- | --- | --- | --- |
| Liver transplant | | | | | | |
| Charlton 2011 | USA | SRTR database 2001–2009 | Retrospective database analysis | Adults (aged ≥18 years) undergoing LT | Total: 35,781  NASH: 1959 (5.5%) | 2009: 9.7% |
| Park 2011 | USA | Transplant Institute at Hawaii Medical Center – East 1998–2009 | Observational cohort study | Patients referred to LT surgeons | Total: 569  NASH: 71 (12.5%) | 2009: 28.6% |
| Singal 2013 | USA | UNOS database 1994–2009 | Retrospective database analysis | Adults (age ≥18 years) undergoing first LT | Total: 54,687  NASH: 1368 (2.5%) | 2009: approximately 8% |
| Wong 2014a | USA | UNOS database 2002–2012 | Retrospective database analysis | Adults (aged ≥18 years) undergoing LT | Total: 41,289  NASH: 7100 (17.2%) | 2002–2012: 13.4% |
| Wong 2014b | USA | UNOS database 2002–2012 | Retrospective database analysis | Adults (aged ≥18 years) undergoing HCC-related LT | Total: 61,868  NASH: 373  Modified NASH: 676 | 2012: 13.5% |
| Singal 2016 | USA | UNOS database 2002–2011 | Retrospective database analysis | Adults (aged ≥18 years) undergoing LT | Total: 38,533  NASH: 3665 (9.5%) | 2011: 14.3% |
| Cholankeril 2017 | USA | UNOS database 2003–2014 | Retrospective database analysis | Adults (aged ≥18 years) undergoing LT | Total: 63,061  NASH: 8266 (13.1%) | 2014: 17.4% |
| Golabi 2018 | USA | SRTR database 1994–2016 | Retrospective database analysis | Adults (aged ≥18 years) listed for LT | Total: 223,391  NASH: 11,598 (5.2%)  NASH + CC: 27,812 (12.4%) | 2016: NASH + CC: 19.5% |
| Haldar 2019 | Europe | ELTR database 2002–2016 | Retrospective database analysis | Adults (aged ≥18 years) undergoing LT | Total: 68,950  NASH: 2741 (4.0%) | 2016: 8.4% |
| Thuluvath 2019a | USA | UNOS database 2002–2016 | Retrospective database analysis | Adults (aged ≥18 years) listed for LT | Total: 33,566  NASH: 7935 (23.6%) | 2016: 16% |
| Younossi 2019 | USA | SRTR database 2002–2016 | Retrospective database analysis | Adults (aged ≥18 years) with HCC listed for LT | Total: 24,431  NASH: 2690 (11.0%) | 2017: 17.9% |
| Henson 2020 | USA | UNOS database 2004–2017 | Retrospective database analysis | First-time adult LT registrants with NASH, HCV, or ALD | Total: 66,077  NASH: 14,197 | Patients with NASH undergoing LT as a proportion of total NASH, ALD, and HCV patients undergoing LT: 21.5% |
| Jothimani 2021 | India | Institute of Liver Disease and Transplantation at Dr. Rela Institute and Medical Centre  2009–2019 | Retrospective observational study | Adults (aged ≥18 years) undergoing LT | Total: 1017  NASH: 396 | 2009–2014: 30.7%  2015–2019: 42.0%^a^ |
| Simultaneous liver and kidney transplant | | | | | | |
| Singal 2013 | USA | UNOS database 1994–2009 | Retrospective database analysis | Adults (age ≥18 years) undergoing first LT | Total: 54,687  NASH: 1368 (2.5%) | 1994–2009: 14% |
| Singal 2014 | USA | UNOS database 2002–2011 | Retrospective database analysis | Adults (aged ≥18 years) receiving simultaneous liver and kidney transplantion | Total: 2606  NASH: 221 | 2011: 15.3% |
| Singal 2016 | USA | UNOS database 2002–2011 | Retrospective database analysis | Adults (aged ≥18 years) undergoing LT | Total: 2162  NASH: 320 | 2011: 14.7% |

ALD, alcoholic liver disease; CC, cryptogenic cirrhosis; ELTR, European Liver Transplant Registry; LT, liver transplantation; N, number; NAFLD, non-alcoholic fatty liver disease; NASH, non-alcoholic steatohepatitis; SRTR; Scientific Registry of Transplant Recipients; UNOS, United Network for Organ Sharing.
*^a^*Significant increase in proportion of patients with NASH compared with 2009–2014 (*P* = .001).

**Table A7.** Key outcomes in included studies

| **Key outcomes** | **Number of publications reporting outcomes** |  |
| --- | --- | --- |
| NASH development from NAFLD or from other diseases | 27 | |
| Disease progression to cirrhosis, HCC, CVD, CRC, or worsening fibrosis | 64 | |
| Complications  Hepatic  Extra-hepatic  Cardiovascular | 39  16  19 | |
| Risk factors associated with the development, presence, progression, or complications of NASH | 52 | |
| NASH reversibility or resolution | 30 | |
| Mortality | 41 | |

CRC, colorectal cancer; CVD, cardiovascular disease; HCC, hepatocellular carcinoma; NAFLD, non-alcoholic fatty liver disease; NASH, non-alcoholic steatohepatitis.

**Table A8.** Quantitative study quality assessment by the Effective Public Health Practice Project

| **Publication** | **Selection bias** | **Study design** | **Confounders** | **Blinding** | **Data collection methods** | **Withdrawals and drop-outs** | **Global rating** |
| --- | --- | --- | --- | --- | --- | --- | --- |
| Yasui 2011 | Moderate | Moderate | Strong | Moderate | Strong | Strong | Strong |
| Singal 2013 | Moderate | Moderate | Strong | Moderate | Strong | Moderate | Strong |
| Singal 2016 | Moderate | Moderate | Strong | Moderate | Strong | Moderate | Strong |
| Wong 2011 | Moderate | Moderate | Strong | Moderate | Strong | Moderate | Strong |
| Young 2020 | Moderate | Moderate | Strong | Moderate | Strong | Moderate | Strong |
| Johnson 2021 | Moderate | Moderate | Strong | N/A | Strong | N/A | Strong |
| Alkhouri 2012 | Moderate | Moderate | Moderate | Moderate | Strong | Strong | Strong |
| Axley 2019 | Moderate | Moderate | Moderate | Moderate | Strong | Strong | Strong |
| Barb 2019 | Moderate | Moderate | Moderate | Moderate | Strong | Strong | Strong |
| Bechmann 2010 | Moderate | Moderate | Moderate | Moderate | Strong | Strong | Strong |
| Bril 2014 | Moderate | Moderate | Moderate | Moderate | Strong | Strong | Strong |
| Chen 2019 | Moderate | Moderate | Moderate | Moderate | Strong | Strong | Strong |
| Eshraghian 2019 | Moderate | Moderate | Moderate | Moderate | Strong | Strong | Strong |
| Fierbinteanu-Braticevici 2011 | Moderate | Moderate | Moderate | Moderate | Strong | Strong | Strong |
| Hashizume 2013 | Moderate | Moderate | Moderate | Moderate | Strong | Strong | Strong |
| Hernandez-Alejandr 2012 | Moderate | Moderate | Moderate | Moderate | Strong | Strong | Strong |
| Kawanaka 2019 | Moderate | Moderate | Moderate | Moderate | Strong | Strong | Strong |
| Kern 2019 | Moderate | Moderate | Moderate | Moderate | Strong | Strong | Strong |
| Piazza 2016 | Moderate | Moderate | Moderate | Moderate | Strong | Strong | Strong |
| Vilar-Gomez 2015 | Moderate | Moderate | Moderate | Moderate | Strong | Strong | Strong |
| Wong 2014b | Moderate | Moderate | Moderate | Moderate | Strong | Strong | Strong |
| Mikolasevic 2021 | Moderate | N/A | N/A | Moderate | Strong | Strong | Strong |
| Bando 2012 | Moderate | Moderate | Moderate | Moderate | Strong | Moderate | Strong |
| Cengiz 2015 | Moderate | Moderate | Moderate | Moderate | Strong | Moderate | Strong |
| Huang 2015 | Moderate | Moderate | Moderate | Moderate | Strong | Moderate | Strong |
| Kennedy 2012 | Moderate | Moderate | Moderate | Moderate | Strong | Moderate | Strong |
| Mahamid 2017 | Moderate | Moderate | Moderate | Moderate | Strong | Moderate | Strong |
| Patel 2018a | Moderate | Moderate | Moderate | Moderate | Strong | Moderate | Strong |
| Pelusi 2019 | Moderate | Moderate | Moderate | Moderate | Strong | Moderate | Strong |
| Shima 2013 | Moderate | Moderate | Moderate | Moderate | Strong | Moderate | Strong |
| Singal 2018 | Moderate | Moderate | Moderate | Moderate | Strong | Moderate | Strong |
| Sourianarayanane 2017a | Moderate | Moderate | Moderate | Moderate | Strong | Moderate | Strong |
| Sourianarayanane 2017b | Moderate | Moderate | Moderate | Moderate | Strong | Moderate | Strong |
| Stine 2015 | Moderate | Moderate | Moderate | Moderate | Strong | Moderate | Strong |
| Stine 2018 | Moderate | Moderate | Moderate | Moderate | Strong | Moderate | Strong |
| Targher 2010 | Moderate | Moderate | Moderate | Moderate | Strong | Moderate | Strong |
| Weingarten 2011 | Moderate | Moderate | Moderate | Moderate | Strong | Moderate | Strong |
| Weinmann 2015 | Moderate | Moderate | Moderate | Moderate | Strong | Moderate | Strong |
| Whitsett 2019 | Moderate | Moderate | Moderate | Moderate | Strong | Moderate | Strong |
| Wong 2014a | Moderate | Moderate | Moderate | Moderate | Strong | Moderate | Strong |
| Wang 2018 | Moderate | Moderate | Moderate | Moderate | Strong | Moderate | Strong |
| Vilar-Gomez 2017 | Moderate | Moderate | Moderate | Moderate | Strong | Moderate | Strong |
| Henson 2020 | Moderate | Moderate | Moderate | Moderate | Moderate | Strong | Strong |
| Kwong 2020 | Moderate | Moderate | Strong | Moderate | Strong | Strong | Moderate |
| Lee 2020 | Moderate | Moderate | Strong | Moderate | Strong | Strong | Moderate |
| Cho 2019 | Strong | Moderate | Strong | Moderate | Strong | Weak | Moderate |
| Vieira Barbosa 2021 | Strong | Moderate | Strong | Weak | Moderate | Strong | Moderate |
| Park 2021 | Moderate | Moderate | Strong | Weak | Strong | Strong | Moderate |
| Ajmera 2018 | Strong | Moderate | Weak | Moderate | Strong | Moderate | Moderate |
| Gill 2011 | Strong | Moderate | Weak | Moderate | Strong | Moderate | Moderate |
| Loomba 2012 | Strong | Moderate | Weak | Moderate | Strong | Moderate | Moderate |
| Michitaka 2010 | Strong | Moderate | Weak | Moderate | Strong | Moderate | Moderate |
| Chisholm 2012 | Moderate | Moderate | Strong | Moderate | Strong | Weak | Moderate |
| D’Ambrosio 2021 | Moderate | Moderate | Strong | Moderate | Strong | Weak | Moderate |
| Holzner 2021 | Moderate | Moderate | Strong | Moderate | Strong | Weak | Moderate |
| Lee HH 2020 | Moderate | Moderate | Strong | Moderate | Strong | Weak | Moderate |
| Eshraghian 2020 | Moderate | Moderate | Strong | Moderate | Strong | Weak | Moderate |
| Vilar-Gomez 2021 | Moderate | Moderate | Strong | Moderate | Weak | Strong | Moderate |
| Yang 2021 | Moderate | Moderate | Strong | Weak | Moderate | Strong | Moderate |
| Kim 2015 | Moderate | Moderate | Moderate | Moderate | Strong | Moderate | Moderate |
| Russo 2021 | Moderate | Moderate | N/A | Weak | Moderate | Strong | Moderate |
| Mazumber 2020 | Moderate | Moderate | Strong | Moderate | Moderate | Weak | Moderate |
| Alkhouri 2010 | Moderate | Moderate | Weak | Moderate | Strong | Moderate | Moderate |
| Anty 2016 | Moderate | Moderate | Weak | Moderate | Strong | Moderate | Moderate |
| Auguet 2014 | Moderate | Moderate | Weak | Moderate | Strong | Moderate | Moderate |
| Asfari 2017 | Moderate | Moderate | Weak | Moderate | Strong | Moderate | Moderate |
| Charlton 2011 | Moderate | Moderate | Weak | Moderate | Strong | Moderate | Moderate |
| Cholankeril 2017 | Moderate | Moderate | Weak | Moderate | Strong | Moderate | Moderate |
| Denkmayr 2018 | Moderate | Moderate | Weak | Moderate | Strong | Moderate | Moderate |
| Doycheva 2019 | Moderate | Moderate | Weak | Moderate | Strong | Moderate | Moderate |
| Fussner 2014 | Moderate | Moderate | Weak | Moderate | Strong | Moderate | Moderate |
| Hermans 2018 | Moderate | Moderate | Weak | Moderate | Strong | Moderate | Moderate |
| Jung 2014 | Moderate | Moderate | Weak | Moderate | Strong | Moderate | Moderate |
| Kakar 2019 | Moderate | Moderate | Weak | Moderate | Strong | Moderate | Moderate |
| Kaswala 2020 | Moderate | Moderate | Weak | Moderate | Strong | Moderate | Moderate |
| Lassailly 2015 | Moderate | Moderate | Weak | Moderate | Strong | Moderate | Moderate |
| Mahamid 2018 | Moderate | Moderate | Weak | Moderate | Strong | Moderate | Moderate |
| Molnar 2019 | Moderate | Moderate | Weak | Moderate | Strong | Moderate | Moderate |
| Nagai 2019 | Moderate | Moderate | Weak | Moderate | Strong | Moderate | Moderate |
| Nilsson 2019 | Moderate | Moderate | Weak | Moderate | Strong | Moderate | Moderate |
| Pagadala 2012 | Moderate | Moderate | Weak | Moderate | Strong | Moderate | Moderate |
| Patel 2018b | Moderate | Moderate | Weak | Moderate | Strong | Moderate | Moderate |
| Pirvulescu 2012 | Moderate | Moderate | Weak | Moderate | Strong | Moderate | Moderate |
| Reddy 2012 | Moderate | Moderate | Weak | Moderate | Strong | Moderate | Moderate |
| Satapathy 2017 | Moderate | Moderate | Weak | Moderate | Strong | Moderate | Moderate |
| Sebastiani 2015 | Moderate | Moderate | Weak | Moderate | Strong | Moderate | Moderate |
| Seko 2015 | Moderate | Moderate | Weak | Moderate | Strong | Moderate | Moderate |
| Sheikh 2012 | Moderate | Moderate | Weak | Moderate | Strong | Moderate | Moderate |
| Sorrentino 2010 | Moderate | Moderate | Weak | Moderate | Strong | Moderate | Moderate |
| Sourianarayanane 2013 | Moderate | Moderate | Weak | Moderate | Strong | Moderate | Moderate |
| Thuluvath 2018a | Moderate | Moderate | Weak | Moderate | Strong | Moderate | Moderate |
| Thuluvath 2019b | Moderate | Moderate | Weak | Moderate | Strong | Moderate | Moderate |
| Thuluvath 2019a | Moderate | Moderate | Weak | Moderate | Strong | Moderate | Moderate |
| Ulitsky 2010 | Moderate | Moderate | Weak | Moderate | Strong | Moderate | Moderate |
| VanWagner 2016 | Moderate | Moderate | Weak | Moderate | Strong | Moderate | Moderate |
| Younossi 2019 | Moderate | Moderate | Weak | Moderate | Strong | Moderate | Moderate |
| Zarrinpar 2019 | Moderate | Moderate | Weak | Moderate | Strong | Moderate | Moderate |
| Choi 2020 | Moderate | Moderate | Moderate | Moderate | Strong | Weak | Moderate |
| Domanski 2012 | Moderate | Moderate | Moderate | Moderate | Strong | Weak | Moderate |
| El Atrache 2012 | Moderate | Moderate | Moderate | Moderate | Strong | Weak | Moderate |
| Heuer 2012 | Moderate | Moderate | Moderate | Moderate | Strong | Weak | Moderate |
| Hindi 2013 | Moderate | Moderate | Moderate | Moderate | Strong | Weak | Moderate |
| Koo 2017 | Moderate | Moderate | Moderate | Moderate | Strong | Weak | Moderate |
| Kruger 2011 | Moderate | Moderate | Moderate | Moderate | Strong | Weak | Moderate |
| Park 2011 | Moderate | Moderate | Moderate | Moderate | Strong | Weak | Moderate |
| Oliveira 2019 | Moderate | Moderate | Moderate | Moderate | Strong | Weak | Moderate |
| Todokai 2019 | Moderate | Moderate | Moderate | Moderate | Strong | Weak | Moderate |
| Vilar-Gomez 2017 | Moderate | Moderate | Moderate | Moderate | Strong | Weak | Moderate |
| Vilar-Gomez 2019 | Moderate | Moderate | Moderate | Moderate | Strong | Weak | Moderate |
| Jothimani 2021 | Moderate | Moderate | N/A | Moderate | Strong | Weak | Moderate |
| Hirose 2020 | Moderate | Moderate | Moderate | Weak | Moderate | Strong | Moderate |
| Reha 2014 | Moderate | Moderate | Weak | Moderate | Moderate | Moderate | Moderate |
| Singh 2010 | Moderate | Moderate | Moderate | Moderate | Weak | Moderate | Moderate |
| Tanaka 2011 | Moderate | Moderate | Moderate | Moderate | Weak | Moderate | Moderate |
| Marti-Aguado 2021 | Moderate | N/A | N/A | Moderate | Moderate | Weak | Moderate |
| Aminian 2021 | Moderate | Moderate | Strong | Weak | Strong | Weak | Weak |
| Ampuero 2021 | Moderate | Moderate | Strong | Weak | Strong | Weak | Weak |
| Billeter 2021 | Moderate | Moderate | Strong | Weak | Strong | Weak | Weak |
| Patel 2021 | Moderate | Moderate | Strong | Weak | Strong | Weak | Weak |
| Rivas 2021 | Moderate | Moderate | Strong | Weak | Strong | Weak | Weak |
| Renno 2021 | Moderate | Moderate | Strong | Weak | Moderate | Weak | Weak |
| Kumar 2020 | Moderate | Moderate | Strong | Moderate | Weak | Weak | Weak |
| Reddy 2020 | Moderate | Moderate | Strong | Moderate | Weak | Weak | Weak |
| Reja 2021 | Weak | Moderate | Strong | Weak | Strong | Weak | Weak |
| Imajo 2014 | Moderate | Moderate | Weak | Weak | Strong | Moderate | Weak |
| Safar 2013 | Moderate | Moderate | Weak | Weak | Strong | Moderate | Weak |
| Al-hamoudi 2020 | Moderate | Moderate | Weak | Moderate | Strong | Weak | Weak |
| Amarapurkar 2013 | Moderate | Moderate | Weak | Moderate | Strong | Weak | Weak |
| Bambha 2012 | Moderate | Moderate | Weak | Moderate | Strong | Weak | Weak |
| Ferslew 2015 | Moderate | Moderate | Weak | Moderate | Strong | Weak | Weak |
| Golabi 2018 | Moderate | Moderate | Weak | Moderate | Strong | Weak | Weak |
| Haldar 2019 | Moderate | Moderate | Weak | Moderate | Strong | Weak | Weak |
| Kimura 2010 | Moderate | Moderate | Weak | Moderate | Strong | Weak | Weak |
| O’Leary 2011 | Moderate | Moderate | Weak | Moderate | Strong | Weak | Weak |
| van den Berg 2018 | Moderate | Moderate | Weak | Moderate | Strong | Weak | Weak |
| VanWagner 2012 | Moderate | Moderate | Weak | Moderate | Strong | Weak | Weak |
| VanWagner 2015 | Moderate | Moderate | Weak | Moderate | Strong | Weak | Weak |
| Ma 2021 | Weak | N/A | N/A | Moderate | Strong | Weak | Weak |
| Nah 2021 | Moderate | N/A | N/A | Weak | Strong | Weak | Weak |
| Angulo 2015 | Moderate | Moderate | Weak | Moderate | Weak | Strong | Weak |
| Ghoneim 2020 | Moderate | Moderate | Weak | Moderate | Weak | Strong | Weak |
| Tan 2021 | Moderate | Moderate | Weak | Weak | Moderate | Strong | Weak |
| Tseng 2021 | Moderate | Moderate | Weak | Weak | Moderate | Strong | Weak |
| Nagai 2021 | Moderate | Moderate | Weak | Weak | Strong | Weak | Weak |
| Pons 2021 | Moderate | Moderate | Weak | Weak | Strong | Weak | Weak |
| Ruiz-Casas 2021 | Moderate | Moderate | N/A | Weak | Moderate | Weak | Weak |
| Francazani 2011 | Moderate | Moderate | Weak | Moderate | Weak | Moderate | Weak |
| GBD 2020 | Moderate | Moderate | Weak | Moderate | Weak | Moderate | Weak |
| Thuluvath 2018b | Moderate | Moderate | Weak | Moderate | Weak | Moderate | Weak |
| Udelsman 2019 | Moderate | Moderate | Weak | Moderate | Weak | Moderate | Weak |
| Karnam 2022 | Moderate | Moderate | N/A | Weak | Weak | Weak | Weak |

N/A, not applicable.

**Table A9.** Quality assessment of included randomized clinical trials

| **Publication** | **Was randomization carried out appropriately?** | **Was the concealment of treatment allocation adequate?** | **Were the groups similar at the outset of the study in terms of prognostic factors?** | **Were the care providers, participants and outcome assessors blind to treatment allocation?** | **Were there any unexpected imbalances in drop-outs between groups?** | **Is there any evidence to suggest that the authors measured more outcomes than they reported?** | **Did the analysis include an ITT analysis? If so, was this appropriate and were appropriate methods used to account for missing data?** |
| --- | --- | --- | --- | --- | --- | --- | --- |
| Cusi 2016 | Yes | Yes | Yes | Yes | No | No | Yes |
| Friedman 2018 | Yes | Yes | Yes | Yes | No | No | Yes |
| Harrison 2021a | Yes | Yes | Yes | Yes | No | No | Yes |
| Harrison 2021b | Yes | Yes | Yes | Yes | No | No | Yes |
| Huang 2021 | Yes | Not clear | Yes | Yes | No | No | Yes |
| Okanoue 2021 | Yes | Not clear | Yes | Yes | No | No | Yes |
| Ratziu 2016 | Yes | Yes | Not clear | Yes | No | No | Yes |
| Promrat 2010 | Yes | Yes | Yes | Yes | No | No | Not clear |
| Newsome 2021 | Yes | Yes | Yes | Yes | No | Yes | Yes |
| Ratziu 2021 | Yes | Yes | Yes | Yes | No | No | No |
| Sanyal 2010 | Yes | Yes | Yes | Yes | Yes | No | Yes |
| Kedarisetty 2021 | Yes | Yes | Yes | No | No | No | Yes |
| Hoofnagle 2013 | Yes | Yes | Yes | Yes | Not clear | No | Not clear |
| Francque 2021 | Yes | Not clear | Yes | Yes | Yes | No | Yes |
| Loomba 2021 | Yes | Yes | Yes | Yes | Yes | No | No |
| Li 2015 | Yes | Not clear | Yes | No | No | No | Not clear |
| Sanyal 2019 | No | Yes | Not clear | Yes | Not clear | No | Yes |
| de Oliveira 2019 | Yes | No | Yes | No | Yes | No | Yes |
| Gurka 2021 | Not clear | Not clear | Yes | Yes | Not clear | No | Not clear |
| Corey 2015 | Not clear | Not clear | Yes | Not clear | Not clear | No | Not clear |
| Corey 2019 | Not clear | Not clear | Not clear | Not clear | Not clear | No | Yes |
| Younossi 2018 | Not clear | Not clear | Not clear | Not clear | Not clear | No | Not clear |

ITT, intent-to-treat.

# Supplementary References

Ajmera V, Belt P, Wilson LA, et al. Among patients with nonalcoholic fatty liver disease, modest alcohol use is associated with less improvement in histologic steatosis and steatohepatitis. Clinical Gastroenterol Hepatol 2018;16:1511–1520.e5.

Al-Hamoudi W, Alsadoon A, Hassanian M, et al. Endothelial dysfunction in nonalcoholic steatohepatitis with low cardiac disease risk. Sci Rep 2020;10:8825.

Alkhouri N, Tamimi TA, Yerian L, et al. The inflamed liver and atherosclerosis: a link between histologic severity of nonalcoholic fatty liver disease and increased cardiovascular risk. Dig Dis Sci 2010;55:2644–2650.

Alkhouri N, Morris-Stiff G, Campbell C, et al. Neutrophil to lymphocyte ratio: a new marker for predicting steatohepatitis and fibrosis in patients with nonalcoholic fatty liver disease. Liver Int 2012;32:297–302.

Amarapurkar DN, Dharod M, Gautam S, et al. Risk of development of hepatocellular carcinoma in patients with NASH-related cirrhosis. Trop Gastroenterol 2013;34:159–163.

Aminian A, Al-Kurd A, Wilson R, et al. Association of bariatric surgery with major adverse liver and cardiovascular outcomes in patients with biopsy-proven nonalcoholic steatohepatitis. JAMA 2021;326:2031–2042.

Ampuero J, Aller R, Gallego-Durán R, et al. Definite and indeterminate nonalcoholic steatohepatitis share similar clinical features and prognosis: a longitudinal study of 1893 biopsy-proven nonalcoholic fatty liver disease subjects. Liver Int 2021;41:2076–2086.

Angulo P, Kleiner DE, Dam-Larsen S, et al. Liver fibrosis, but no other histologic features, is associated with long-term outcomes of patients with nonalcoholic fatty liver disease. Gastroenterology 2015;149:389–397.e10.

Anty R, Hastier A, Canivet CM, et al. Severe vitamin D deficiency is not associated with liver damage in morbidly obese patients. Obes Surg 2016;26:2138–2143.

Asfari MM, Niyazi F, Lopez R, et al. The association of nonalcoholic steatohepatitis and obstructive sleep apnea. Eur J Gastroenterol Hepatol 2017;29:1380–1384.

Auguet T, Berlanga A, Guiu-Jurado E, et al. Altered fatty acid metabolism-related gene expression in liver from morbidly obese women with non-alcoholic fatty liver disease. Int J Mol Sci 2014;15:22173–22187.

Axley P, Ahmed Z, Arora S, et al. NASH is the most rapidly growing etiology for acute-on-chronic liver failure-related hospitalization and disease burden in the United States: a population-based study. Liver Transpl 2019;25:695–705.

Bambha K, Belt P, Abraham M, et al. Ethnicity and nonalcoholic fatty liver disease. Hepatology 2012;55:769–780.

Bando Y, Kanehara H, Aoki K, et al. The glycated albumin to glycated haemoglobin ratio increases along with the fibrosis stage in non-alcoholic steatohepatitis. Ann Clin Biochem 2012;49:387–390.

Barb D, Bril F, Kalavalapalli S, et al. Plasma fibroblast growth factor 21 is associated with severity of nonalcoholic steatohepatitis in patients with obesity and type 2 diabetes. J Clin Endocrinol Metab 2019;104:3327–3336.

Bechmann LP, Gieseler RK, Sowa JP, et al. Apoptosis is associated with CD36/fatty acid translocase upregulation in non-alcoholic steatohepatitis. Liver International 2010;30:850–859.

Billeter AT, Müller PC, Albrecht T, et al. Impact of type 2 diabetes on oncologic outcomes of hepatocellular carcinomas in non-cirrhotic, non-alcoholic steatohepatitis: a matched-pair analysis. J Gastrointest Surg 2021;25:1193–1202.

Bril F, Lomonaco R, Orsak B, et al. Relationship between disease severity, hyperinsulinemia, and impaired insulin clearance in patients with nonalcoholic steatohepatitis. Hepatology 2014;59:2178–2187.

Cengiz M, Ozenirler S, Yılmaz G, et al. Impact of hepatic immunoreactivity of angiotensin-converting enzyme 2 on liver fibrosis due to non-alcoholic steatohepatitis. Clin Res Hepatol Gastroenterol 2015;39:692–698.

Charlton MR, Burns JM, Pedersen RA, et al. Frequency and outcomes of liver transplantation for nonalcoholic steatohepatitis in the United States. Gastroenterology 2011;141:1249–1253.

Chen J, Zheng M, Luo Y, et al. Ratio of conjugated chenodeoxycholic to muricholic acids is associated with severity of nonalcoholic steatohepatitis. Obesity (Silver Spring) 2019;27:2055–2066.

Chisholm J, Seki Y, Toouli J, et al. Serologic predictors of nonalcoholic steatohepatitis in a population undergoing bariatric surgery. Surg Obes Relat Dis 2012;8:416–422.

Cho Y, Lim SK, Joo SK, et al. Nonalcoholic steatohepatitis is associated with a higher risk of advanced colorectal neoplasm. Liver Int 2019;39:1722–1731.

Choi HSJ, Brouwer WP, Zanjir WMR, et al. Nonalcoholic steatohepatitis is associated with liver-related outcomes and all-cause mortality in chronic hepatitis B. Hepatology 2020;71:539–548.

Cholankeril G, Wong RJ, Hu M, et al. Liver transplantation for nonalcoholic steatohepatitis in the US: temporal trends and outcomes. Dig Dis Sci 2017;62:2915–2922.

Corey KE, Wilson LA, Altinbas A, et al. Relationship between resolution of non-alcoholic steatohepatitis and changes in lipoprotein sub-fractions: a post-hoc analysis of the PIVENS trial. Aliment Pharmacol Ther 2019;49:1205–1213.

Corey KE, Vuppalanchi R, Wilson LA, et al. NASH resolution is associated with improvements in HDL and triglyceride levels but not improvement in LDL or non-HDL-C levels. Aliment Pharmacol Ther 2015;41:301–309.

Cusi K, Orsak B, Bril F, et al. Long-term pioglitazone treatment for patients with nonalcoholic steatohepatitis and prediabetes or type 2 diabetes mellitus: a randomized trial. Ann Intern Med 2016;165:305–315.

D'Ambrosio R, Campi I, Maggioni M, et al. The relationship between liver histology and thyroid function tests in patients with non-alcoholic fatty liver disease (NAFLD). PLoS One 2021;16:e0249614.

de Oliveira DG, de Faria Ghetti F, Moreira APB, et al. Association between dietary total antioxidant capacity and hepatocellular ballooning in nonalcoholic steatohepatitis: a cross-sectional study. Eur J Nutr 2019;58:2263–2270.

Denkmayr L, Feldman A, Stechemesser L, et al. Lean patients with non-alcoholic fatty liver disease have a severe histological phenotype similar to obese patients. J Clin Med 2018;7:562.

Domanski JP, Park SJ, Harrison SA. Cardiovascular disease and nonalcoholic fatty liver disease: does histologic severity matter? J Clin Gastroenterol 2012;46:427–430.

Doycheva I, Zhang T, Amjad W, et al. Diabetes and hepatocellular carcinoma: incidence trends and impact of liver disease etiology. J Clin Exp Hepatol 2019;10:296–303.

El Atrache MM, Abouljoud MS, Divine G, et al. Recurrence of non-alcoholic steatohepatitis and cryptogenic cirrhosis following orthotopic liver transplantation in the context of the metabolic syndrome. Clin Transpl 2012;26:E505–E512.

Eshraghian A, Nikeghbalian S, Dehghani M, et al. Nonalcoholic steatohepatitis is the most rapidly growing indication for liver transplantation in Iranian patients. Exp Clin Transplant 2022;20:487–494.

Eshraghian A, Nikeghbalian S, Geramizadeh B, et al. Characterization of biopsy proven non-alcoholic fatty liver disease in healthy non-obese and lean population of living liver donors: the impact of uric acid. Clin Res Hepatol Gastroenterol 2020;44:572–578.

Ferslew BC, Xie G, Johnston CK, et al. Altered bile acid metabolome in patients with nonalcoholic steatohepatitis. Dig Dis Sci 2015;60:3318–3328.

Fierbinteanu-Braticevici C, Baicus C, Tribus L, et al. Predictive factors for nonalcoholic steatohepatitis (NASH) in patients with nonalcoholic fatty liver disease (NAFLD). J Gastrointestin Liver Dis 2011;20:153–159.

Fracanzani AL, Valenti L, Bugianesi E, et al. Risk of nonalcoholic steatohepatitis and fibrosis in patients with nonalcoholic fatty liver disease and low visceral adiposity. J Hepatol 2011;54:1244–1249.

Francque SM, Bedossa P, Ratziu V, et al. A randomized, controlled trial of the pan-PPAR agonist lanifibranor in NASH. N Eng J Med 2021;385:1547–1558.

Friedman SL, Ratziu V, Harrison SA, et al. A randomized, placebo-controlled trial of cenicriviroc for treatment of nonalcoholic steatohepatitis with fibrosis. Hepatology 2018;67:1754–1767.

Fussner LA, Charlton MR, Heimbach JK, et al. The impact of gender and NASH on chronic kidney disease before and after liver transplantation. Liver Int 2014;34:1259–1266.

GBD 2017 Cirrhosis Collaborators. The global, regional, and national burden of cirrhosis by cause in 195 countries and territories, 1990–2017: a systematic analysis for the Global Burden of Disease Study 2017. Lancet Gastroenterol Hepatol 2020;5:245–266.

Ghoneim S, Butt MU, Hamid O, et al. The incidence of COVID-19 in patients with metabolic syndrome and non-alcoholic steatohepatitis: a population-based study. Metabol Open 2020;8:100057.

Gill RM, Belt P, Wilson L, et al. Centrizonal arteries and microvessels in nonalcoholic steatohepatitis. Am J Surg Pathol 2011;35:1400–1404.

Golabi P, Bush H, Stepanova M, et al. Liver transplantation (LT) for cryptogenic cirrhosis (CC) and nonalcoholic steatohepatitis (NASH) cirrhosis: data from the Scientific Registry of Transplant Recipients (SRTR): 1994 to 2016. Medicine (Baltimore) 2018;97:e11518.

Gurka MJ, Mack JA, Chi X, et al. Use of metabolic syndrome severity to assess treatment with vitamin E and pioglitazone for non-alcoholic steatohepatitis. J Gastroenterol Hepatol 2021;36:249–256.

Haldar D, Kern B, Hodson J, et al. Outcomes of liver transplantation for non-alcoholic steatohepatitis: a European Liver Transplant Registry study. J Hepatol 2019;71:313–322.

Harrison SA, Neff G, Guy CD, et al. Efficacy and safety of aldafermin, an engineered FGF19 analog, in a randomized, double-blind, placebo-controlled trial of patients with nonalcoholic steatohepatitis. Gastroenterology 2021a;160:219–231.e1.

Harrison SA, Ruane PJ, Freilich BL, et al. Efruxifermin in non-alcoholic steatohepatitis: a randomized, double-blind, placebo-controlled, phase 2a trial. Nat Med 2021b;27:1262–1271.

Hashizume H, Sato K, Yamazaki Y, et al. A prospective study of long-term outcomes in female patients with nonalcoholic steatohepatitis using age- and body mass index-matched cohorts. Acta Med Okayama 2013;67:45–53.

Henson JB, Wilder JM, Kappus MR, et al. Transplant outcomes in older patients with nonalcoholic steatohepatitis compared to alcohol-related liver disease and hepatitis C. Transplantation 2020;104:e164–e173.

Hermans MP, Dath N. Prevalence and co-prevalence of comorbidities in Belgian patients with type 2 diabetes mellitus: a transversal, descriptive study. Acta Clin Belg 2018;73:68–74.

Hernandez-Alejandro R, Croome KP, Drage M, et al. A comparison of survival and pathologic features of non-alcoholic steatohepatitis and hepatitis C virus patients with hepatocellular carcinoma. World J Gastroenterol 2012;18:4145–4149.

Heuer M, Kaiser GM, Kahraman A, et al. Liver transplantation in nonalcoholic steatohepatitis is associated with high mortality and post-transplant complications: a single-center experience. Digestion 2012;86:107–113.

Hindi M, Levy C, Couto CA, et al. Primary biliary cirrhosis is more severe in overweight patients. J Clin Gastroenterol 2013;47:e28–32.

Hirose S, Matsumoto K, Tatemichi M, et al. Nineteen-year prognosis in Japanese patients with biopsy-proven nonalcoholic fatty liver disease: lean versus overweight patients. PLoS One 2020;15:e0241770.

Holzner ML, Florman S, Schwartz ME, et al. Outcomes of liver transplantation for nonalcoholic steatohepatitis-associated hepatocellular carcinoma. HPB (Oxford) 2022;24:470–477.

Hoofnagle JH, Van Natta ML, Kleiner DE, et al. Vitamin E and changes in serum alanine aminotransferase levels in patients with non-alcoholic steatohepatitis. Aliment Pharmacol Ther 2013;38:134–143.

Huang JF, Yeh ML, Yu ML, et al. Hyperuricemia inversely correlates with disease severity in Taiwanese nonalcoholic steatohepatitis patients. PLoS One 2015;10:e0139796.

Huang JF, Dai CY, Huang CF, et al. First-in-Asian double-blind randomized trial to assess the efficacy and safety of insulin sensitizer in nonalcoholic steatohepatitis patients. Hepatol Int 2021;15:1136–1147.

Imajo K, Hyogo H, Yoneda M, et al. LDL-migration index (LDL-MI), an indicator of small dense low-density lipoprotein (sdLDL), is higher in non-alcoholic steatohepatitis than in non-alcoholic fatty liver: a multicenter cross-sectional study. PLoS One 2014;9:e115403.

Johnson PC, Cochet AA, Gore RS, et al. Early cardiac dysfunction in biopsy-proven nonalcoholic fatty liver disease. Korean J Gastroenterol 2021;78:161–167.

Jothimani D, Danielraj S, Narasimhan G, et al. Nonalcoholic steatohepatitis: a rapidly increasing indication for liver transplantation in India. J Clin Exp Hepatol 2022;12:908–916.

Jung KY, Cho SY, Kim HJ, et al. Nonalcoholic steatohepatitis associated with metabolic syndrome: relationship to insulin resistance and liver histology. J Clin Gastroenterol 2014;48:883–888.

Kakar S, Dugum M, Cabello R, et al. Incidence of recurrent NASH-related allograft cirrhosis. Dig Dis Sci 2019;64:1356–1363.

Karnam RS, Mitsakakis N, Saracino G, et al. Predicting long-term survival after liver transplantation in patients with NASH cirrhosis. Clin Gastroenterol Hepatol 2022;20:704–705.

Kaswala DH, Zhang J, Liu A, et al. A comprehensive analysis of liver transplantation outcomes among ethnic minorities in the United States. J Clin Gastroenterol 2020;54:263–270.

Kawanaka M, Tanikawa T, Kamada T, et al. High prevalence of autoimmune gastritis in patients with nonalcoholic steatohepatitis. Intern Med 2019;58:2907–2913.

Kedarisetty CK, Bhardwaj A, Kumar G, et al. Efficacy of combining pentoxiphylline and vitamin E versus vitamin E alone in non-alcoholic steatohepatitis– a randomized pilot study. Indian J Gastroenterol 2021;40:41–49.

Kennedy C, Redden D, Gray S, et al. Equivalent survival following liver transplantation in patients with non-alcoholic steatohepatitis compared with patients with other liver diseases. HPB (Oxford) 2012;14:625–634.

Kern B, Feurstein B, Fritz J, et al. High incidence of hepatocellular carcinoma and postoperative complications in patients with nonalcoholic steatohepatitis as a primary indication for deceased liver transplantation. Eur J Gastroenterol Hepatol 2019;31:205–210.

Kim G, Lee YH, Park YM, et al. Use of a diabetes self-assessment score to predict nonalcoholic fatty liver disease and nonalcoholic steatohepatitis. Medicine (Baltimore) 2015;94:e1103.

Kimura Y, Hyogo H, Yamagishi S, et al. Atorvastatin decreases serum levels of advanced glycation endproducts (AGEs) in nonalcoholic steatohepatitis (NASH) patients with dyslipidemia: clinical usefulness of AGEs as a biomarker for the attenuation of NASH. J Gastroenterol 2010;45:750–757.

Koo BK, Kim D, Joo SK, et al. Sarcopenia is an independent risk factor for non-alcoholic steatohepatitis and significant fibrosis. J Hepatol 2017;66:123–131.

Kruger FC, Daniels CR, Kidd M, et al. APRI: a simple bedside marker for advanced fibrosis that can avoid liver biopsy in patients with NAFLD/NASH. S Afr Med J 2011;101:477–480.

Kumar R, Goh BG, Kam JW, et al. Comparisons between non-alcoholic steatohepatitis and alcohol-related hepatocellular carcinoma. Clin Mol Hepatol 2020;26:196–208.

Kwong AJ, Devuni D, Wang C, et al. Outcomes of liver transplantation among older recipients with nonalcoholic steatohepatitis in a large multicenter US cohort: the Re-evaluating Age Limits in Transplantation consortium. Liver Transpl 2020;26:1492–1503.

Lassailly G, Caiazzo R, Buob D, et al. Bariatric surgery reduces features of nonalcoholic steatohepatitis in morbidly obese patients. Gastroenterology 2015;149:379–388.

Lee HH, Cho Y, Choi YJ, et al. Non-alcoholic steatohepatitis and progression of carotid atherosclerosis in patients with type 2 diabetes: a Korean cohort study. Cardiovasc Diabetol 2020a;19:81.

Lee H, Kim G, Choi YJ, et al. Association between non-alcoholic steatohepatitis and left ventricular diastolic dysfunction in type 2 diabetes mellitus. Diabetes Metab J 2020b;44:267–276.

Li YH, Yang LH, Sha KH, et al. Efficacy of poly-unsaturated fatty acid therapy on patients with nonalcoholic steatohepatitis. World J Gastroenterol 2015;21:7008–7013.

Loomba R, Abraham M, Unalp A, et al. Association between diabetes, family history of diabetes, and risk of nonalcoholic steatohepatitis and fibrosis. Hepatology 2012;56:943–951.

Loomba R, Noureddin M, Kowdley KV, et al. Combination therapies including cilofexor and firsocostat for bridging fibrosis and cirrhosis attributable to NASH. Hepatology 2021;73:625–643.

Ma HL, Zheng KI, Rios RS, et al. Histological characteristics of non-alcoholic steatohepatitis in NAFLD patients with low degree of hepatocyte apoptosis. Turk J Gastroenterol 2021;32:758–764.

Mahamid M, Yassin T, Abu Elheja O, et al. Association between fatty liver disease and hyperplastic colonic polyp. Isr Med Assoc J 2017;19:105–108.

Mahamid M, Mahroum N, Bragazzi NL, et al. Folate and B12 levels correlate with histological severity in NASH patients. Nutrients 2018;10:440.

Marti-Aguado D, Rodríguez-Ortega A, Mestre-Alagarda C, et al. Digital pathology: accurate technique for quantitative assessment of histological features in metabolic-associated fatty liver disease. Aliment Pharmacol Ther 2021;53:160–171.

Mazumder NR, Celaj S, Atiemo K, et al. Liver-related mortality is similar among men and women with cirrhosis. J Hepatol 2020;73:1072–1081.

Michitaka K, Nishiguchi S, Aoyagi Y, et al. Etiology of liver cirrhosis in Japan: a nationwide survey. J Gastroenterol 2010;45:86–94.

Mikolasevic I, Delija B, Mijic A, et al. Small intestinal bacterial overgrowth and non-alcoholic fatty liver disease diagnosed by transient elastography and liver biopsy. Int J Clin Pract 2021;75:e13947.

Molnar MZ, Joglekar K, Jiang Y, et al. Association of pretransplant renal function with liver graft and patient survival after liver transplantation in patients with nonalcoholic steatohepatitis. Liver Transplant 2019;25:399–410.

Nagai S, Collins K, Chau LC, et al. Increased risk of death in first year after liver transplantation among patients with nonalcoholic steatohepatitis vs liver disease of other etiologies. Clin Gastroenterol Hepatol 2019;17:2759–2768.e5.

Nagai S, Safwan M, Kitajima T, et al. Disease-specific waitlist outcomes in liver transplantation – a retrospective study. Transpl Int 2021;34:499–513.

Nah EH, Cho S, Park H, et al. Subclinical steatohepatitis and advanced liver fibrosis in health examinees with nonalcoholic fatty liver disease (NAFLD) in 10 South Korean cities: a retrospective cross-sectional study. PLoS One 2021;16:e0260477.

Newsome PN, Buchholtz K, Cusi K, et al. A placebo-controlled trial of subcutaneous semaglutide in nonalcoholic steatohepatitis. N Eng J Med 2021;384:1113–1124.

Nilsson E, Anderson H, Sargenti K, et al. Clinical course and mortality by etiology of liver cirrhosis in Sweden: a population based, long-term follow-up study of 1317 patients. Aliment Pharmacol Ther 2019;49:1421–1430.

Okanoue T, Sakamoto M, Harada K, et al. Efficacy and safety of apararenone (MT-3995) in patients with nonalcoholic steatohepatitis: a randomized controlled study. Hepatol Res 2021;51:943–956.

O'Leary JG, Landaverde C, Jennings L, et al. Patients with NASH and cryptogenic cirrhosis are less likely than those with hepatitis C to receive liver transplants. Clin Gastroenterol Hepatol 2011;9:700–704.e1.

Oliveira CP, Cotrim HP, Stefano JT, et al. N-acetylcysteine and/or ursodeoxycholic acid associated with metformin in non-alcoholic steatohepatitis: an open-label multicenter randomized controlled trial. Arq Gastroenterol 2019;56:184–190.

Pagadala MR, Zein CO, Dasarathy S, et al. Prevalence of hypothyroidism in nonalcoholic fatty liver disease. Dig Dis Sci 2012;57:528–534.

Park CW, Tsai NT, Wong LL. Implications of worse renal dysfunction and medical comorbidities in patients with NASH undergoing liver transplant evaluation: impact on MELD and more. Clin Transplant 2011;25:E606–E611.

Park JH, Koo BK, Kim W, et al. Histological severity of nonalcoholic fatty liver disease is associated with 10-year risk for atherosclerotic cardiovascular disease. Hepatol Int 2021;15:1148–1159.

Patel YA, Gifford EJ, Glass LM, et al. Risk factors for biopsy-proven advanced non-alcoholic fatty liver disease in the Veterans Health Administration. Aliment Pharmacol Ther 2018b;47:268–278.

Patel SS, Nabi E, Guzman L, et al. Coronary artery disease in decompensated patients undergoing liver transplantation evaluation. Liver Transpl 2018a;24:333–342.

Patel S, Siddiqui MB, Chandrakumaran A, et al. Progression to cirrhosis leads to improvement in atherogenic milieu. Dig Dis Sci 2021;66:263–272.

Pelusi S, Cespiati A, Rametta R, et al. Prevalence and risk factors of significant fibrosis in patients with nonalcoholic fatty liver without steatohepatitis. Clin Gastroenterol Hepatol 2019;17:2310–2319.e6.

Piazza NA, Singal AK. Frequency of cardiovascular events and effect on survival in liver transplant recipients for cirrhosis due to alcoholic or nonalcoholic steatohepatitis. Exp Clin Transplant 2016;14:79–85.

Pirvulescu I, Gheorghe L, Csiki I, et al. Noninvasive clinical model for the diagnosis of nonalcoholic steatohepatitis in overweight and morbidly obese patients undergoing bariatric surgery. Chirurgia (Bucur) 2012;107:772–779.

Pons M, Augustin S, Scheiner B, et al. Noninvasive diagnosis of portal hypertension in patients with compensated advanced chronic liver disease. Am J Gastroenterol 2021;116:723–732.

Promrat K, Kleiner DE, Niemeier HM, et al. Randomized controlled trial testing the effects of weight loss on nonalcoholic steatohepatitis. Hepatology 2010;51:121–129.

Ratziu V, Harrison SA, Francque S, et al. Elafibranor, an agonist of the peroxisome proliferator-activated receptor-α and -δ, induces resolution of nonalcoholic steatohepatitis without fibrosis worsening. Gastroenterology 2016;150:1147–1159.e5.

Ratziu V, de Guevara L, Safadi R, et al. Aramchol in patients with nonalcoholic steatohepatitis: a randomized, double-blind, placebo-controlled phase 2b trial. Nat Med 2021;27:1825–1835.

Reddy SK, Steel JL, Chen HW, et al. Outcomes of curative treatment for hepatocellular cancer in nonalcoholic steatohepatitis versus hepatitis C and alcoholic liver disease. Hepatology 2012;55:1809–1819.

Reddy YK, Marella HK, Jiang Y, et al. Natural history of non-alcoholic fatty liver disease: a study with paired liver biopsies. J Clin Exp Hepatol 2020;10:245–254.

Reha JL, Lee S, Hofmann LJ. Prevalence and predictors of nonalcoholic steatohepatitis in obese patients undergoing bariatric surgery: a Department of Defense experience. Am Surg 2014;80:595–599.

Reja M, Patel R, Pioppo L, et al. Renal failure is associated with increased mortality and hospital utilization in patients admitted with nonalcoholic steatohepatitis. J Clin Gastroenterol 2021;55:433–438.

Renno A, Abdel-Aziz Y, Alastal Y, et al. The association between obstructive sleep apnea and non-alcoholic steatohepatitis: a retrospective nationwide inpatient sample analysis. Clin Exp Hepatol 2021;7:25–29.

Rivas G, Hummer-Bair B, Bezinover D, et al. Plasminogen activator inhibitor is significantly elevated in liver transplant recipients with decompensated NASH cirrhosis. BMJ Open Gastroenterol 2021;8:e000683.

Ruiz-Casas L, Pedra G, Shaikh A, et al. Clinical and sociodemographic determinants of disease progression in patients with nonalcoholic steatohepatitis in the United States. Medicine (Baltimore) 2021;100:e28165.

Russo MF, Lembo E, Mari A, et al. Insulin resistance is central to long-term reversal of histologic nonalcoholic steatohepatitis after metabolic surgery. J Clin Endocrinol Metab 2021;106:750–761.

Safar Zadeh E, Lungu AO, Cochran EK, et al. The liver diseases of lipodystrophy: the long-term effect of leptin treatment. J Hepatol 2013;59:131–137.

Sanyal AJ, Chalasani N, Kowdley KV, et al. Pioglitazone, vitamin E, or placebo for nonalcoholic steatohepatitis. N Engl J Med 2010;362:1675–1685.

Sanyal AJ, Harrison SA, Ratziu V, et al. The natural history of advanced fibrosis due to nonalcoholic steatohepatitis: data from the simtuzumab trials. Hepatology 2019;70:1913–1927.

Satapathy SK, Jiang Y, Eason JD, et al. Cardiovascular mortality among liver transplant recipients with nonalcoholic steatohepatitis in the United States-a retrospective study. Transpl Int 2017;30:1051–1060.

Sebastiani G, Alshaalan R, Wong P, et al. Prognostic value of non-invasive fibrosis and steatosis tools, hepatic venous pressure gradient (HVPG) and histology in nonalcoholic steatohepatitis. PLoS One 2015;10:e0128774.

Seko Y, Sumida Y, Tanaka S, et al. Serum alanine aminotransferase predicts the histological course of non-alcoholic steatohepatitis in Japanese patients. Hepatol Res 2015;45:E53–E61.

Sheikh MY, Raoufi R, Atla PR, et al. Prevalence of cirrhosis in patients with thrombocytopenia who receive bone marrow biopsy. Saudi J Gastroenterol 2012;18:257–262.

Shima T, Uto H, Ueki K, et al. Clinicopathological features of liver injury in patients with type 2 diabetes mellitus and comparative study of histologically proven nonalcoholic fatty liver diseases with or without type 2 diabetes mellitus. J Gastroenterol 2013;48:515–525.

Singal AK, Guturu P, Hmoud B, et al. Evolving frequency and outcomes of liver transplantation based on etiology of liver disease. Transplantation 2013;95:755–760.

Singal AK, Salameh H, Kuo YF, et al. Evolving frequency and outcomes of simultaneous liver kidney transplants based on liver disease etiology. Transplantation 2014;98:216–221.

Singal AK, Hasanin M, Kaif M, et al. Nonalcoholic steatohepatitis is the most rapidly growing indication for simultaneous liver kidney transplantation in the United States. Transplantation 2016;100:607–612.

Singh DK, Rastogi A, Sakhuja P, et al. Comparison of clinical, biochemical and histological features of alcoholic steatohepatitis and non-alcoholic steatohepatitis in Asian Indian patients. Indian J Pathol Microbiol 2010;53:408–413.

Sorrentino P, Terracciano L, D'Angelo S, et al. Predicting fibrosis worsening in obese patients with NASH through parenchymal fibronectin, HOMA-IR, and hypertension. Am J Gastroenterol 2010;105:336–344.

Sourianarayanane A, O'Shea RS, Barnes DS, et al. High prevalence of normal serum albumin in NASH patients with ascites: a retrospective analysis. Clin Res Hepatol Gastroenterol 2013;37:246–253.

Sourianarayanane A, Arikapudi S, McCullough AJ, et al. Nonalcoholic steatohepatitis recurrence and rate of fibrosis progression following liver transplantation. Eur J Gastroenterol Hepatol 2017a;29:481–487.

Sourianarayanane A, Talluri J, Humar A, et al. Stage of fibrosis and portal pressure correlation in nonalcoholic steatohepatitis. Eur J Gastroenterol Hepatol 2017b;29:516–523.

Stine JG, Shah NL, Argo CK, et al. Increased risk of portal vein thrombosis in patients with cirrhosis due to nonalcoholic steatohepatitis. Liver Transpl 2015;21:1016–1021.

Stine JG, Niccum BA, Zimmet AN, et al. Increased risk of venous thromboembolism in hospitalized patients with cirrhosis due to non-alcoholic steatohepatitis. Clin Transl Gastroenterol 2018;9:140.

Tan HK, Teng MLP, Soh AYS, et al. Poor outcomes of cirrhosis due to nonalcoholic steatohepatitis compared with hepatitis B after decompensation with ascites. Am J Gastroenterol 2021;116:1437–1446.

Tanaka N, Horiuchi A, Yokoyama T, et al. Clinical characteristics of de novo nonalcoholic fatty liver disease following pancreaticoduodenectomy. J Gastroenterol 2011;46:758–768.

Targher G, Bertolini L, Rodella S, et al. Relationship between kidney function and liver histology in subjects with nonalcoholic steatohepatitis. Clin J Am Soc Nephrol 2010;5:2166–2171.

Thuluvath PJ, Hanish S, Savva Y. Liver transplantation in cryptogenic cirrhosis: outcome comparisons between NASH, alcoholic, and AIH cirrhosis. Transplantation 2018a;102:656–663.

Thuluvath PJ, Kantsevoy S, Thuluvath AJ, et al. Is cryptogenic cirrhosis different from NASH cirrhosis? J Hepatol 2018b;68:519–525.

Thuluvath AJ, Chen PH, Thuluvath PJ, et al. Poor survival after retransplantation in NASH cirrhosis. Transplantation 2019b;103:101–108.

Thuluvath PJ, Hanish S, Savva Y. Waiting list mortality and transplant rates for NASH cirrhosis when compared with cryptogenic, alcoholic, or AIH cirrhosis. Transplantation 2019a;103:113–121.

Tokodai K, Karadagi A, Kjaernet F, et al. Characteristics and risk factors for recurrence of nonalcoholic steatohepatitis following liver transplantation. Scand J Gastroenterol 2019;54:233–239.

Tseng J, Korman J, Noureddin M, et al. Routine versus selective liver biopsy during bariatric surgery: postoperative outcomes and preoperative predictors of NASH. Obes Surg 2021;32:463–471.

Udelsman BV, Corey KE, Lindvall C, et al. Risk factors and prevalence of liver disease in review of 2557 routine liver biopsies performed during bariatric surgery. Surg Obes Relat Dis 2019;15:843–849.

Ulitsky A, Ananthakrishnan AN, Komorowski R, et al. A noninvasive clinical scoring model predicts risk of nonalcoholic steatohepatitis in morbidly obese patients. Obesity Surgery 2010;20:685–691.

van den Berg EH, Douwes RM, de Meijer VE, et al. Liver transplantation for NASH cirrhosis is not performed at the expense of major post-operative morbidity. Dig Liver Dis 2018;50:68–75.

VanWagner LB, Bhave M, Te HS, et al. Patients transplanted for nonalcoholic steatohepatitis are at increased risk for postoperative cardiovascular events. Hepatology 2012;56:1741–1750.

VanWagner LB, Lapin B, Skaro AI, et al. Impact of renal impairment on cardiovascular disease mortality after liver transplantation for nonalcoholic steatohepatitis cirrhosis. Liver Int 2015;35:2575–2583.

VanWagner LB, Serper M, Kang R, et al. Factors associated with major adverse cardiovascular events after liver transplantation among a national sample. Am J Transplant 2016;16:2684–2694.

Vieira Barbosa J, Milligan S, Frick A, et al. Fibrosis-4 index as an independent predictor of mortality and liver-related outcomes in NAFLD. Hepatol Commun 2022;6:765–779.

Vilar-Gomez E, Martinez-Perez Y, Calzadilla-Bertot L, et al. Weight loss through lifestyle modification significantly reduces features of nonalcoholic steatohepatitis. Gastroenterology 2015;149:367–378.e5.

Vilar-Gomez E, Calzadilla-Bertot L, Friedman SL, et al. Serum biomarkers can predict a change in liver fibrosis 1 year after lifestyle intervention for biopsy-proven NASH. Liver Int 2017;37:1887–1896.

Vilar-Gomez E, Vuppalanchi R, Desai AP, et al. Long-term metformin use may improve clinical outcomes in diabetic patients with non-alcoholic steatohepatitis and bridging fibrosis or compensated cirrhosis. Aliment Pharmacol Ther 2019;50:317–328.

Vilar-Gomez E, Vuppalanchi R, Gawrieh S, et al. Vitamin E improves transplant-free survival and hepatic decompensation among patients with nonalcoholic steatohepatitis and advanced fibrosis. Hepatology 2020;71:495–509.

Vilar-Gomez E, Calzadilla-Bertot L, Wong VW, et al. Type 2 diabetes and metformin use associate with outcomes of patients with nonalcoholic steatohepatitis-related, Child-Pugh A cirrhosis. Clin Gastroenterol Hepatol 2021;19:136–145.e6.

Wang D, Wang L, Wang Z, et al. Higher non-HDL-cholesterol to HDL-cholesterol ratio linked with increased nonalcoholic steatohepatitis. Lipids Health Dis 2018;17:67.

Weingarten TN, Swain JM, Kendrick ML, et al. Nonalcoholic steatohepatitis (NASH) does not increase complications after laparoscopic bariatric surgery. Obes Surg 2011;1:1714–1720.

Weinmann A, Alt Y, Koch S, et al. Treatment and survival of non-alcoholic steatohepatitis associated hepatocellular carcinoma. BMC Cancer 2015;15:210.

Whitsett M, Wilcox J, Yang A, et al. Atrial fibrillation is highly prevalent yet undertreated in patients with biopsy-proven nonalcoholic steatohepatitis. Liver Int 2019;39:933–940.

Wong VW, Wong GL, Tsang SW, et al. High prevalence of colorectal neoplasm in patients with non-alcoholic steatohepatitis. Gut 2011;60:829–836.

Wong RJ, Chou C, Bonham CA, et al. Improved survival outcomes in patients with non-alcoholic steatohepatitis and alcoholic liver disease following liver transplantation: an analysis of 2002–2012 United Network for Organ Sharing data. Clin Transplant 2014a;28:713–721.

Wong RJ, Cheung R, Ahmed A. Nonalcoholic steatohepatitis is the most rapidly growing indication for liver transplantation in patients with hepatocellular carcinoma in the U.S. Hepatology 2014b;59:2188–2195.

Yang RX, Zou ZS, Zhong BH, et al. The pathologic relevance of metabolic criteria in patients with biopsy-proven nonalcoholic fatty liver disease and metabolic dysfunction associated fatty liver disease: a multicenter cross-sectional study in China. Hepatobiliary Pancreat Dis Int 2021;20:426–432.

Yasui K, Sumida Y, Mori Y, et al. Nonalcoholic steatohepatitis and increased risk of chronic kidney disease. Metabolism 2011;60:735–739.

Young S, Sanghvi T, Rubin N, et al. Transarterial chemoembolization of hepatocellular carcinoma: propensity score matching study comparing survival and complications in patients with nonalcoholic steatohepatitis versus other causes cirrhosis. Cardiovasc Intervent Radiol 2020;43:65–75.

Younossi Z, Stepanova M, Sanyal AJ, et al. The conundrum of cryptogenic cirrhosis: adverse outcomes without treatment options. J Hepatol 2018;69:1365–1370.

Younossi Z, Stepanova M, Ong JP, et al. Nonalcoholic steatohepatitis is the fastest growing cause of hepatocellular carcinoma in liver transplant candidates. Clin Gastroenterol Hepatol 2019;17:748–755.e3.

Zarrinpar A, Faltermeier CM, Agopian VG, et al. Metabolic factors affecting hepatocellular carcinoma in steatohepatitis. Liver Int 2019;39:531–539.
